# Machine learning and metagenomics identifies uncharacterized taxa inferred to drive biogeochemical cycles in a subtropical hypereutrophic estuary

**DOI:** 10.1093/ismeco/ycae067

**Published:** 2024-05-10

**Authors:** Apoorva Prabhu, Sanjana Tule, Maria Chuvochina, Mikael Bodén, Simon J McIlroy, Julian Zaugg, Christian Rinke

**Affiliations:** School of Chemistry and Molecular Biosciences, Australian Centre for Ecogenomics, The University of Queensland, QLD 4072, Australia; School of Chemistry and Molecular Biosciences, The University of Queensland, QLD 4072, Australia; School of Chemistry and Molecular Biosciences, Australian Centre for Ecogenomics, The University of Queensland, QLD 4072, Australia; School of Chemistry and Molecular Biosciences, The University of Queensland, QLD 4072, Australia; Centre for Microbiome Research, School of Biomedical Sciences, Translational Research Institute, Queensland University of Technology, QLD 4102, Australia; School of Chemistry and Molecular Biosciences, Australian Centre for Ecogenomics, The University of Queensland, QLD 4072, Australia; School of Chemistry and Molecular Biosciences, Australian Centre for Ecogenomics, The University of Queensland, QLD 4072, Australia; Department of Microbiology, University of Innsbruck, 6020 Innsbruck, Austria

**Keywords:** hypereutrophic estuary, MAGs, machine learning, uncharacterised taxa, nutrient cycling, carbon cycling

## Abstract

Anthropogenic influences have drastically increased nutrient concentrations in many estuaries globally, and microbial communities have adapted to the resulting hypereutrophic ecosystems. However, our knowledge of the dominant microbial taxa and their potential functions in these ecosystems has remained sparse. Here, we study prokaryotic community dynamics in a temporal–spatial dataset, from a subtropical hypereutrophic estuary. Screening 54 water samples across brackish to marine sites revealed that nutrient concentrations and salinity best explained spatial community variations, whereas temperature and dissolved oxygen likely drive seasonal shifts. By combining short and long read sequencing data, we recovered 2,459 metagenome-assembled genomes, proposed new taxon names for previously uncharacterised lineages, and created an extensive, habitat specific genome reference database. Community profiling based on this genome reference database revealed a diverse prokaryotic community comprising 61 bacterial and 18 archaeal phyla, and resulted in an improved taxonomic resolution at lower ranks down to genus level. We found that the vast majority (61 out of 73) of abundant genera (>1% average) represented unnamed and novel lineages, and that all genera could be clearly separated into brackish and marine ecotypes with inferred habitat specific functions. Applying supervised machine learning and metabolic reconstruction, we identified several microbial indicator taxa responding directly or indirectly to elevated nitrate and total phosphorus concentrations. In conclusion, our analysis highlights the importance of improved taxonomic resolution, sheds light on the role of previously uncharacterised lineages in estuarine nutrient cycling, and identifies microbial indicators for nutrient levels crucial in estuary health assessments.

## Introduction

Estuaries are dynamic ecosystems that facilitate the exchange of dissolved and particulate organic and inorganic matter from marine, terrestrial and freshwater biomes [1–3]. Cycling of carbon, derived from organic matter (OM), and nutrients is enhanced in estuaries due to tidal influences that increase water circulation and prolong water residence times [4, 5]. Sustained population growth and rapid economic development have exposed many rivers and estuaries to anthropogenic pressures [6], which resulted in increased levels of OM and nutrients, specifically nitrogen and phosphorus, from sources such as wastewater effluent and agricultural runoff [7].

Microbial communities play a crucial role in estuary ecosystems since they drive the main biogeochemical processes, such as carbon, nitrogen, phosphorus, and sulphur cycling [8, 9]. Identifying key microbial taxa that mediate these cycles is essential for our comprehension of nutrient fate and transport in these dynamic ecosystems, including nutrient flux to coastal environments [8, 10]. However, our understanding of the diversity and function of estuary microbes is limited. This is especially true for subtropical and tropical estuaries, since only a few studies have employed metagenomics to explore their microbial communities [11–14].

Profiling of the dominant microbial taxa in subtropical and tropical, eutrophic estuaries has mainly been restricted to higher taxonomic ranks, including the phyla *Pseudomonadota* (former Proteobacteria), *Bacteroidetes*, *Actinobacteria*, *Cyanobacteria*, and *Verrucomicrobia* [11, 12, 15, 16], and rarely resolves taxa at lower ranks, e.g. genus. Increasing the taxonomic resolution of microbial community profiles is essential to gain key ecological insights, and will, in combination with the identification of microbial indicator taxa that are indicative of distinct nutrient concentrations (nitrates and total phosphorus [TP]), allow us to establish microbiological criteria for monitoring estuarine ecosystems [17]. Particularly elevated nutrients levels, which are characteristic for many eutrophic estuaries, have been linked to microbial nitrogen uptake and remineralisation, such as nitrification [13], dissimilatory nitrate reduction (DNRA) [18, 19], and denitrification [9]. In hypereutrophic waters, DNRA and denitrification were found to be active processes even under fully oxygenated conditions [20]. Studies on the microbial taxa carrying out these processes in tropical, euphoric estuaries have remained rare. An investigation of the nitrogen cycle in the subtropical Pearl and Yangtze River estuaries, inferred that ammonia oxidising archaea (AOA) assigned to *Nitrososphaeria* and the nitrite oxidizer *Nitrospira* were driving the nitrification process [13, 21]. Microbial taxa involved in phosphorus cycling in the water column of tropical and subtropical eutrophic estuaries have not been explored, which is surprising given that elevated phosphorus levels from anthropogenic sources, such as sewage or from dredging sediment, are main contributors to eutrophication in aquatic ecosystems [22, 23]. Sulphur cycling has been reported to be mediated by members of the classes *Alpha-* and *Gammaproteobacteria*, and *Cyanobacteria* in the Pearl River estuary [15, 16]. High levels of organic carbon in the Pearl River estuary have been linked to microbial carbon degradation genes such as pectins, xylan, and peptidoglycan, although the taxa carrying these genes have not been identified [15]. However, bacteria associated with inorganic carbon fixation in this estuary have been reported to include the phyla *Cyanobacteriota*, *Nitrospinota*, and *Bacillota* [15].

Studying microbial communities in the Brisbane River estuary, provides an opportunity to expand our knowledge of microbial taxa and their roles in nutrient cycling (carbon, nitrogen, phosphorus, and sulphur) in subtropical, hypereutrophic estuaries. Nutrient levels in the Brisbane River have increased considerably due to anthropogenic influences, resulting in high export rates of phosphorus (685 t/year) and nitrogen (3162 t/year), which are estimated to be 5.3 and 2.7 times greater than before European settlement [24]. Severe alteration of the river bed, which are common anthropogenic impacts in subtropical estuaries [25, 26], have also shaped the Brisbane River. For example, decades of dredging have increased the impact of tidal currents that resuspend fine-grained sediment from the river bed, causing a markedly higher turbidity [27, 28] that is responsible for the murky, brown colour of the estuary and the high sediment export rates [29]. So far, microbial communities in the Brisbane River estuary have only been assessed by 16S ribosomal ribonucleic acid (rRNA) gene sequencing [30] and are still awaiting more comprehensive profiling with metagenomic sequencing.

Here, we apply a genome-focused metagenomics approach, supported by machine learning (ML), to dissect microbial communities in the subtropical Brisbane River estuary. The recovery of nearly 2,500 metagenome-assembled genomes (MAGs) improved the resolution of our prokaryotic community profiles and revealed spatial and temporal patterns on the genus level in response to changes in physicochemical conditions. We characterised brackish and marine ecotypes, defined as a set of taxa using similar ecological niches, and inferred their roles in biogeochemical cycling in the estuary. We subsequently opted to take full advantage of ML models that provide an ensemble of decision trees and can capture our observed nonlinear relationships more effectively than linear statistical models. This study is therefore, a first attempt to identify significant ecological indicator taxa by applying an ML approach, consisting of Indicator Value (IndVal) analyses (IVA) followed by a random forest ML classifier.

## Materials and methods

### Sample collection and processing

**Figure 1:**
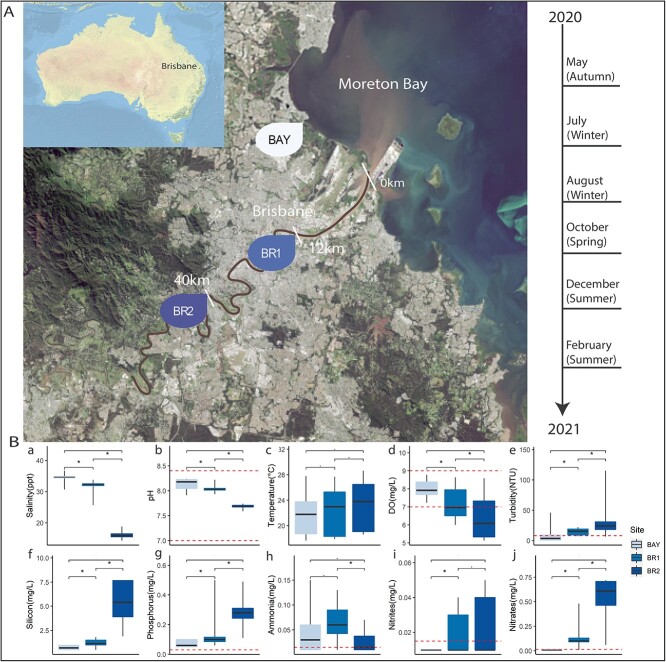
Spatial and temporal survey of the Brisbane River Estuary. A) Map of Australia depicting the city of Brisbane (inset), and an enlarged satellite image of the sampling sites in the Brisbane River Estuary, comprising the sites BR1 and BR2, located 12 and 40 km upstream from the river mouth, respectively, and site BAY, located in Moreton Bay 9 km from the river mouth. The sampling timeline is provided on the right. The path of the lower Brisbane River is hightlighted for visibility. B) Corresponding physico-chemical parameters collected from each site are displayed on the right, and parameters that are significantly different, between all three sites, are marked with an asterisk (*). Dashed lines indicate threshold values for the Brisbane River estuary given by DES, (Queensland Department of Environment and Science) [46] which are, for example 0.2 mg/L for nitrates and 0.02 mg/L for total phosphorus (TP) whereby measurements exceeding these limits indicate hypereutrophication. Credits: A) image courtesy of NASA images 2017 (https://landsat.visibleearth.nasa.gov/view.php?id=85828).

Surface water samples were collected from three sites along the lower Brisbane River estuary (BR1 and BR2) and Moreton Bay (BAY) (Fig. 1A). In total, 54 water samples were collected, by sampling in triplicates every 2 months (May 2020–February 2021) with at least one time point for each season (summer and winter = 2 sampling trips; autumn and spring = 1 sampling trip; Fig. 1A) at high tide. Additional sampling trips were carried out in February, and in October for BR1 and BR2 sites at low tide. Around ~7 L of water was transported back into the laboratory and processed within 4–12 h of collection. The samples were first prefiltered using 8 um 142 mm filters fitted into Merck filtration towers and cells collected onto 0.22 um Sterivex filters. DNA was extracted from the filters using a modified phenol chloroform extraction method (See Supplementary Text) yielding ~1–2 ug of DNA for metagenomics sequencing. Physical and chemical parameters (Table S1) were analysed by Horiba U-52G (Japan) and by ALS Global, Brisbane.

### Metagenomic sequencing and phylogenetic analyses

Metagenome libraries were prepared using Nextera Flex (Illumina, UA) library kits and sequenced on Illumina NovaSeq 6000 (Ramaciotti Genomics, Sydney) using 150 bp paired-end reads that yielded 8–10 GB per library (Table S2). Oxford Nanopore long reads were generated from the BAY and BR1 samples using a single library (see Materials and methods), and sequenced on PromethION 24 for 72 h on R9.4 flow cell using MinKNOW (v20.06.18) with default settings. Subsequently, Illumina reads were assembled using metaSPAdes v3.15.0 [31], and the assembled scaffolds (Table S3) were binned using Aviary (v0.6.0 unpublished, R. Newell, https://github.com/rhysnewell/aviary) using differential coverage information from the same sampling month as the assembled scaffolds (i.e. all reads from each sampling month) to generate MAGs (Table S4). Additional MAGs were also generated from two additional datasets—pilot sample data in February 2020 for all the three sites and during low tide in October 2020 from the two estuary sites (BR1 and BR2) (Table S4). Nanopore sequencing yielded 40–70 Gbps of data that was subsequently basecalled using Guppy (v4.0.11). Long reads were assembled with their corresponding short reads to create hybrid assemblies, followed by binning with Metabat2 v2.12.1 (see Supplementary Text), using the slamM assembly and binning pipeline (https://github.com/Ecogenomics/slamM). All MAGs were dereplicated at 99% ANI using Galah “cluster” (v.0.3.0, unpublished, B. Woodcroft https://github.com/wwood/galah), taking into account CheckM [32] (v1.1.3) quality scores. The 99% ANI dereplication cutoff was selected to exclude nearly identical genomes whilst still allowing the recovery of interspecies diversity. MAGs were assigned a taxonomy using the Genome Taxonomy Database (GTDB) Toolkit (GTDB-tk; v1.6.0) with reference to GTDB (GTDB R207, [33]). MAGs that were of medium (MQ) and high quality (HQ) based on MIMAG [34] criteria were selected for further analyses. Dereplicated hybrid assembly MAGs were screened for adapters using Porechop (v.0.2.4, unpublished, R. Wick, https://github.com/rrwick/Porechop), and contigs with middle adapters identified were discarded using the discard middle flag. MAG quality scores were recalculated after contig removal, and only MAGs with an estimated minimum completeness of >50% and a maximum contamination of <10% were retained. Relative abundances for the MAGs were calculated by mapping Illumina reads to MAGs and species representatives from GTDB using CoverM (v0.6.1, unpublished, B. Woodcroft, unpublished, https://github.com/wwood/CoverM) at 95% percent identity and 75% alignment thresholds.

A bootstrapped phylogenetic tree (B = 100) was constructed by running FastTree2 [35] (v2.1.9) for the bacterial domain. A FastTree was also inferred for Archaea and used as a starting tree, followed by IQ-TREE 2 (v 2.1.2, [35, 37] to infer a maximum likelihood phylogeny using the LG + C10 + F + G model. Rank normalisation for the bacterial and archaeal tree was carried out using PhyloRank (v0.0.37, D. Parks, unpublished, https://github.com/dparks1134/PhyloRank/), which calculates a relative evolutionary divergence (RED value) for each taxon in a phylogenetic tree. Next, several rounds of manual curation, each of which was followed by PhyloRank-based rank evaluations, allowed to refine taxon placements within the phylogenetic trees. The resulting trees were visualised in iTOL v6 (https://itol.embl.de/). The novel families and genera identified were named “NF__” and “NG__”, respectively, throughout the manuscript. Names were proposed for a subset of these novel taxa and with the recent development of the SeqCode [38, 39] new taxa can now be proposed and validated based on DNA sequences, including metagenome assembled genomes (MAGs), as type material [40]. For community profiling, a new database was created with singleM (v0.13.2dev unpublished, B. Woodcroft, https://wwood.github.io/singlem/) using “supplement” flag, which supplements genomes recovered from this study into existing singleM metapackage (S3.1.0.metapackage_20221209.smpkg.zb) against GTDB. To generate a taxonomic profile to include newly named families and genera, all Illumina reads were first screened for quality using FastQC v0.11.9 for quality check and were processed through singleM with “p” flag (see Supplementary Text) which clusters reads to operational taxonomic units (OTUs) using 37 bacterial and 35 archaeal single-copy marker genes using taxonomies from GTDB.

### Gene and genome centric analyses

For gene centric analyses, a non-redundant gene catalogue for all samples was first constructed by gene calling all assembled scaffolds using Prodigal [41], extracting complete proteins (and corresponding nucleotide sequences) and clustering with CD-HIT [42] at 90% similarity with at least 80% coverage. The resultant nonredundant proteins were then annotated using DRAM v(v1.3) [43]. Reads were mapped to the corresponding nucleotide sequences using coverM at 95% percent identity and 75% alignment thresholds to generate a coverage table, which was linked to the corresponding annotations (KEGG, Pfam, CAZy, VOGDB, and hypothetical proteins). Only genes with KEGG and CAZy annotations were explored for the biogeochemical pathways, as they represented approximately 65% of the non-redundant annotated gene catalogue. Genes with significant differential abundance were analysed using the Log2FoldChange (1 > x < 1) and *P*-value cutoffs (*P* < .001). Gene calling was done on all MAGs using Prodigal and annotations checked against DRAM and METABOLIC-G [44] (v4.0) databases. Habitat specific functions were inferred by comparing differential abundances of key genes in nutrient cycling pathways. Thereby, we focused on carbon fixation and degradation, nitrogen, phosphorus, and sulphur cycling. For example, the BAY site had higher relative abundance of photosynthesis and nitrogen assimilation genes, whereas the brackish site BR2 had higher relative abundance of nitrogen removal genes such as denitrification and DNRA.

### Sequence analyses

Alpha diversity was measured using Shannon diversity index and was computed from nonrarefied genus level OTU tables generated from singleM output using *phyloseq* (v1.42.0), *breakaway* (v4.8.4), and *DivNet* (v.0.4.0) packages in R v4.2.2. Significance testing was done using permutational analysis of variance (PERMANOVA) using the *adonis* function in vegan R package (v.2.6.4) with Bray–Curtis distance metric and with a nonparametric Tukey test. Environmental factors influencing alpha diversity of communities were fitted into a linear regression model (lm) to check for significance. Beta diversity patterns and influence of physico-chemical properties across sites and seasons were visualised with Principal component analyses (PCA) using the *pca* function and unconstrained non-metric multidimensional scaling (NMDS) and fitting the environmental vectors into the ordination using the envfit function (*vegan* package v2.6.4) in R. Biplots of PCA were plotted using the *stats* (v3.6.2) package in R. OTU abundance was normalised using the variance stabilising transformation (vst) function in DESeq2 (v1.38.3) package, and the 50 most abundant OTUs across sites and seasons were selected for differential analyses, which filtered OTUs based on Log2FoldChange (1 > x < 1) and *P*-value cutoffs (*P* < .001). Pairwise permutational (PERMANOVA) with Benjamini–Hochberg corrections were carried out to test for statistically significant variance amongst multivariate microbial community data using the *pairwiseAdonis* (v.0.3) function in the *vegan* package. Correlations between environmental variables were assessed using Spearman’s rank correlation tests through the rcorr function (*P*-values <0.05 were considered significant) in the *hmisc* (v4.7) package in R.

### Definition of brackish and marine ecotypes

We adopted the definition of an ecotype that is commonly used in microbial ecology, i.e. a group of taxa, or organisms, that co-occur spatially and/or temporally and are therefore associated with similar environmental conditions [36]. Based on our community profiles and by including all OTUs, we established two ecotypes, marine and brackish. Marine ecotypes were required to have higher average relative abundances, across the entire time series and all replicates, at the marine site BAY compared to the brackish site BR2. Contrarily, brackish ecotypes were defined based on higher average abundances at the brackish site BR2. For the genome centric analysis, the relative abundances were established by stringent metagenomic read mapping, using CoverM at 95% percent identity and 75% alignment thresholds, against all recovered MAGs and 6,009 family level representative genomes (one representative genome for each family; *see supplementary text Materials and methods, Suppl. File GTDB rep per rank family bacteria*) from GTDB. The ecotypes were then established following the same criteria that were applied to the community profiles.

### IndVal analyses and random forest ML approach

Following an initial exploration using a regression analysis, it became evident that this method was not suitable for our data (see supplementary text). We therefore opted for a classification based approach, i.e.fitting a machine-learning (ML), that captured our observed non-linear relationships more effectively than linear statistical models. Overall, our ML input data were features and classes, OTU’s relative abundances was then used as input i.e. the features, for our workflow. Classes were defined by creating three categories for nitrate and total phosphorus (TP) measurements, respectively. The detailed ML workflow, consisting of three main blocks and was designed as follows: In the first “data preprocessing” block the data was prepared for the building and training of ML models. We first pre-filter OTUs with counts ≤10 across all 54 samples in the time series, to exclude low abundance OTUs with very few reads per sample. Next, we also identified significant indicator taxa (OTUs) using IVA (*indicspecies* package v1.7.13) with the following thresholds: 1000 permutations, minimum specificity (At) and sensitivity (Bt) set to 70% and *P*-value ≤.001 for nitrates, and ≤.01 for TP. Lastly, we categorise nitrate and TP concentration into three classes based on the variation from the threshold using a custom python script (v3.6.9). The nitrate concentration was divided into normal (<0.015 mg/L), elevated (0.015 mg/L < × > 0.3 mg/L), and high (≥0.3 mg/L). For TP, we divided the classes into elevated (0.03 mg/L > × < 0.1 mg/L), high (0.1 mg/L ≥ × < 0.15 mg/L), and severe (≥0.15 mg/L). In the next “feature selection/model training block”, we trained two different classifiers—random forest and k-nearest neighbours (KNN) on the features selected by Boruta (https://github.com/scikit-learn-contrib/boruta_py) and SelectKbest (feature selection algorithms) respectively. Random forest, KNN, and SelectKBest methods were obtained from the scikit-learn ML python library [45]. We performed cross validation using repeated stratified k-fold cross-validation with 10-folds and 5 repetitions to estimate the capability of both classifiers. In the last “model interpretation” block, we used the SHapely Additive exPlanations (SHAP) library to explain the output of the best performing classifier.

## Results

### Environmental characteristics confirm a structured but dynamic hyper-eutrophic ecosystem

A total of 54 samples were collected over a 1-year time period from three sampling sites in the Brisbane River estuary, i.e. the brackish sites termed “BR1” and “BR2” and the marine site “BAY” (Fig. 1A). The recorded physico-chemical parameters were well above the water quality thresholds issued by the Queensland Government [46] and confirmed a hypereutrophic, brackish to marine ecosystem with considerably elevated levels of ammonia, total phosphorus (TP), and nitrates across all sites (Fig. 1B; g, h, and j). Regardless of these consistently high nutrient loads, we observed pronounced spatial and temporal variations in physico-chemical measurements (Fig. S1). For example, salinity, pH and dissolved oxygen (DO) were significantly higher in the BAY (*P* < .001) compared to BR2, whereas the reverse was true for nutrient (silicon, TP, and nitrates; *P* < .001) and turbidity levels (Fig. S1; A to J). Seasonal variations included, increased turbidity and ammonia levels in spring and summer, respectively (Fig. S1; O and R), higher water temperatures, nitrite levels, as well as decreased DO availability, in summer compared to winter (Fig. S1; M, N, and S, Table S1).

### Genome recovery reveals taxonomic novelty in most lineages

**Figure 2:**
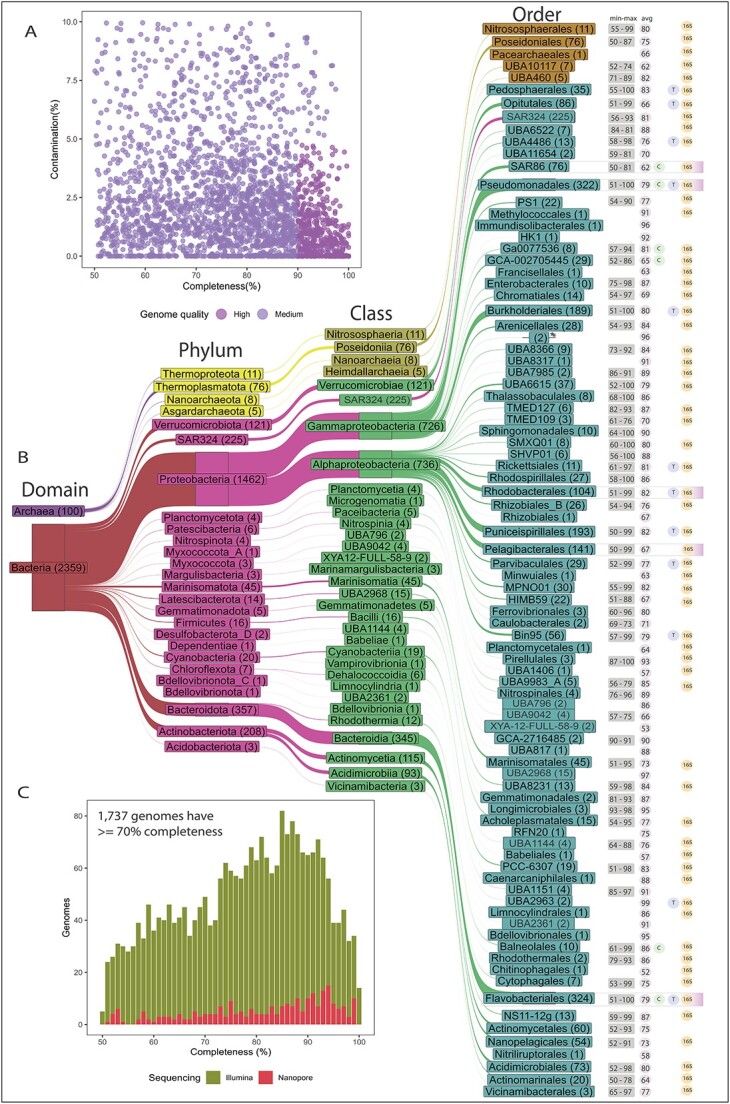
Genome recovery and taxonomic novelty of the recovered MAGs. A) Plot comparing estimated completeness and estimated contamination of all medium quality (MQ) and high quality (HQ) MAGs. MQ and HQ MAGs are defined as having an estimated completeness >50% and 90%, respectively, and an estimated contamination <10% and 5%, respectively. B) Sankey’s plot depicting the taxonomy of MAGs from phylum to order, for archaea and bacteria. The number of MAGs recovered for each taxon is provided in brackets. For each order, minimum, maximum and average estimated completeness are provided. Orders of ecological relevance, which are detailed in the results, are highlighted in the figure as coloured bars in the rightmost column. C) Comparison of MAG qualities between Illumina short read and Oxford Nanopore long read sequencing derived MAGs. ^*^Denotes novel orders. Please note that the homotypic synonym “Proteobacteria” is used for the phylum *Pseudomonadota*.

Assembly, binning and dereplication of metagenomic data yielded 2,459 MAGs, whereby 1,986 and 473 MAGs were categorised as MQ and HQ genomes, respectively (Fig. 2A and B, Table S4), according to MIMAG standards [34]. The average estimated completeness across all MAGs was 78.81 +/− 3.70%, and more than two-thirds had >70% completeness (Fig. 2A, Table S4). Most MAGs (2,210) were recovered from Illumina shotgun read assemblies (Fig. 2C). Both ONT and Illumina data were available for a subset of samples, allowing hybrid assemblies, which added another 249 MAGs (Table S4). Out of these hybrid assembly MAGs, 17 consisted of a single contig, of which nine were predicted to be circular with estimated genome sizes ranging from 1.04 to 2.66 Mbp (Table S5, Fig. 2B). Initial taxonomic assignments were refined by phylogenomic inferences based on 120 and 53 GTDB marker proteins for *Bacteria* and *Archaea*, respectively. Phylogenomic trees were decorated with the GTDB taxonomy and rank normalised, based on relative evolutionary divergence (RED), resulting in the assignment of our MAGs to 22 bacterial (Fig. S2) and 4 archaeal phyla (Fig. 2B, Fig. S3, Table S4). The majority of MAGs were assigned to the phylum *Pseudomonadota* (homotypic synonym “Proteobacteria”; 1462 MAGs), followed by *Bacteroidota* (357), *Actinobacteriota* (208), and *Verrucomicrobiota* (121). The nine circular hybrid assembly MAGs were assigned to uncharacterised taxa (genus to order) in the phyla Proteobacteria (7 MAGs) and *Bacteroidota* (2 MAGs) (Table S5). Overall, only ~10% of MAGs (257), assigned to four archaeal and 15 bacterial phyla, contained a near full copy (>1200 bp) of the 16S rRNA gene (Fig. 2B, Table S6).

Initial community profiles were recovered with a single-copy marker gene analysis (see Materials and methods) and allowed us to identify the most abundant genera in our dataset (Tables S7–S9), the majority of which were only assigned non-Latin placeholder names, hereafter referred to as “uncharacterized taxa”, in the GTDB R207 (Table S10). For six of these genera, our MAGs represented the highest quality genome and qualified as type material [47]. In addition, rank normalisation, based on relative evolutionary divergence identified four novel families and 49 novel genera, which were not present in the GTDB R207 (Table S7, see supplementary text). From this analysis, MAGs of five novel genera, also fulfilled the type material requirements [47]. All 11 genera, i.e. 5 novel and 6 uncharacterized taxa, were placed on stable phylogenetic nodes with 100% bootstrap support, by inferring order or class levels trees (Figs. S4–S7). We propose names for these 11 genera and their higher ranks (8 families and 3 orders), belonging to four bacterial phyla (Table 1, Table S10). We also propose a new species name within the genus *Nitrosopumilus* (Table 1, Fig. S5; A).

**Table 1:** Proposed names for bacterial genera and associated higher ranks. The criterion for MAGs to be assigned to the same species is defined as ANI >95%. Novel genera recovered in this study are indicated by “n.a. (novel)” in the former name column. ^*^This MAG has 6% contamination, which is primarily attributable to 83% strain heterogeneity (see supplementary text). Abbreviations: former GTDB placeholder name = former name; assembly size (million base pairs) = size (Mbp); estimated completeness (%) = com; estimated contamination (%) = cont; 16S rRNA gene length (base pairs) = 16S (bp); MAGs assigned to this species = #MAGs.

GTDB taxonomy (parent taxa)	Proposed genus and species name	Former name	Type genome: size (Mbp), com, cont, 16S (bp), (#MAGs), NCBI Accession
**p__Bacteroidota;c__Bacteroidia**			
o__Flavobacteriales; f__Flavobacteriaceae	*Eutrophosalina*	MED-G14	1.5Mbp; 96%; 0%; 1522 (#11) JAYQTD000000000
o__Flavobacteriales; f__Flavobacteriaceae;g__Eutrophosalina	*Eutrophosalina marina*	.	
**p__Chloroflexota;c__Dehalococcoidia**			
o__UBA2963;f__UBA2963;	*Australimonas*	n.a. (novel)	1.9 Mbp; 97%; 0%; 1472 bp (#1) JAYQTA000000000
o__UBA2963;f__UBA2963; g__Australimonas	*Australimonas brisbanensis*		
**p__Proteobacteria;c__Alphaproteobacteria**			
o__Pelagibacterales;f__Pelagibacteraceae	*Hypereutrophica*	SYDM01	0.9 Mbp; 92%; 6%^*^; 1468 bp (#32) JAYQTL000000000
o__Pelagibacterales;f__Pelagibacteraceae; g__Hypereutrophica	*Hypereutrophica brisbanensis*		
o__Puniceispirillales;f__AAA536-G10	*Marisalimonas*	AAA536-G10	1.9 Mbp; 91%; 0.1%; 1482 bp (#8) JAYPPH000000000
o__Puniceispirillales;f__AAA536-G10; g__Marisalimonas	*Marisalimonas marina*		
o__Rhodobacterales;f__Rhodobacteraceae	*Salinivivens*	MED-G52	2.6 Mbp; 94%; 0.3%; 1457 bp (#9) JAYQKI000000000
o__Rhodobacterales;f__Rhodobacteraceae; g__Salinivivens	*Salinivivens marinus*		
**p__Proteobacteria;c__Gammaproteobacteria**			
o__Burkholderiales;f__UBA3031	*Eutrophomonas*	n.a. (novel)	2.1 Mbp; 98%; 0%; 1532 bp (#39) JAYQIQ000000000
o__Burkholderiales;f__UBA3031; g__Eutrophomonas	*Eutrophomonas brisbanensis*		
o__Burkholderiales;f__UBA3031	*Eutrophovita*	n.a. (novel)	2.1 Mbp; 93%; 0.6%; 1532 bp (#30) JAYQTM000000000
o__Burkholderiales;f__UBA3031; g__Eutrophovita	*Eutrophovita brisbanensis*		
o__Pseudomonadales;f__HTCC2089	*Salivita*	RGAU01	3.3 Mbp; 91%; 1.4%; 1532 bp (#1) JAYQNS000000000
o__Pseudomonadales;f__HTCC2089; g__Salivita	*Salivita marina*		
o__Pseudomonadales;f__Pseudohongiellaceae	*Eutrophocola*	RFVC01	3 Mbp; 94%; 1.8%; 1551 bp (#3) JAYQTB000000000
o__Pseudomonadales;f__Pseudohongiellaceae; g__Eutrophocola	*Eutrophocola salsuginis*		
o__UBA4486;f__UBA4486	*Eutrophobius*	n.a. (novel)	2.7 Mbp; 98%; 0.8%; 1540 bp (#9) JAYPQE000000000
o__UBA4486;f__UBA4486; g__Eutrophobius	*Eutrophobius brisbanensis*		
**p__Thermoproteota; c__Nitrososphaeria**			
o__Nitrososphaerales; f__Nitrosopumilaceae; g__Nitrosopumilus	*Nitrosopumilus brisbanensis*	n.a. (novel)	1.3 Mbp; 93%; 1%; 1422 bp (#2) JAYOXR000000000
**p__Verrucomicrobiota;c__Verrucomicrobia**			
o__Opitutales;f__Opitutaceae	*Salsuginivita*	CAIYFD01	2.4 Mbp; 99%; 0%; 1555 bp (#1) JAYQOU000000000
o__Opitutales;f__Opitutaceae; g__Salsuginivita	*Salsuginivita brisbanensis*		
**Proposed names for higher taxonomic ranks**			
**GTDB Taxonomy (higher taxa)**	**Proposed family/order name**	**Former name**	**Type genus**
**p__Chloroflexota**			
c__Dehalococcoidia; o__UBA2963	*Australimonadaceae*	f__UBA2963	*Australimonas*
c__Dehalococcoidia	*Australimonadales*	o__UBA2963	*Australimonas*
**p__Proteobacteria; c__Alphaproteobacteria**			
o__Puniceispirillales	*Marisalimonadaceae*	f__AAA536-G10	*Marisalimonas*
**p__Proteobacteria; c__Gammaproteobacteria**			
o__Burkholderiales	*Eutrophomonadaceae*	f__UBA3031	*Eutrophomonas*
o__Burkholderiales	*Eutrophovitaceae*	f__UBA3031	*Eutrophovita*
o__Pseudomonadales	*Salivitaceae*	f__HTCC2089	*Salivita*
o__UBA4486	*Eutrophobiaceae*	f__UBA4486	*Eutrophobius*
c__Gammaproteobacteria	*Eutrophobiales*	o__UBA4486	*Eutrophobius*

### Spatial and temporal shifts in microbial diversity are linked to environmental parameters

**Figure 3:**
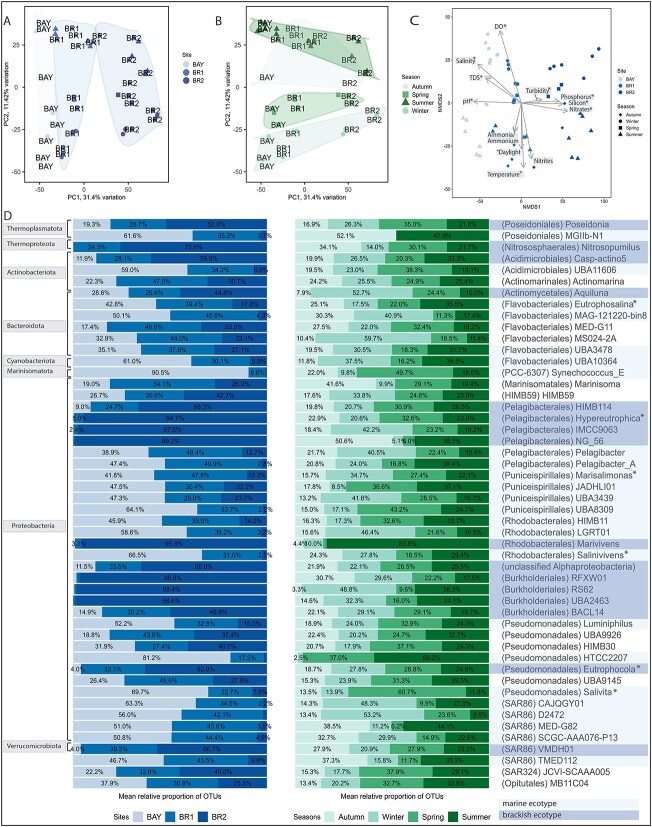
Spatial and temporal trends of the 50 most abundant genera in the Brisbane River estuary. A) Principal component analysis (PCA) of prokaryotic community profiles coloured by site. B) PCA of prokaryotic community profiles coloured by season. C) Unconstrained non-metric multidimensional scaling (NMDS) indicating environmental drivers of the prokaryotic communities. Significant drivers are marked with an asterisk (*). D) Shown are the 50 most abundant OTUs across sites and seasons, defined at the genus level, cumulatively representing >77% of the community by relative abundance. Mean relative proportions of OTUs across sites and seasons were calculated for each of the 50 OTUs. For each genus level OTU, the higher ranks phylum and order are provided in square brackets to the left of the barplot and in curved brackets to the left of the genus name, respectively. Selected genera, which were classified as marine or brackish ecotypes based on significant differential abundance (p_adj_ < 0.05) between the sites BAY and BR2, are highlighted in the rightmost column. The figure includes the newly proposed genus names *Eutrophomonas* (MED-G14), *Hypereutrophica* (SYDM01), *Marisalimonas* (AAA536-G10), *Salinivivens* (MED-G52), *Eutrophocola* (RGAU01), and *Salivita* (RFVC01). Note that Synechococcus_E has been updated to *Parasynechococcus* in GTDB R214. Please note that the homotypic synonym “Proteobacteria” is used for the phylum *Pseudomonadota*.

Prokaryotic community profiles were generated using a single-copy marker gene dataset that included all MAGs recovered in this study (see Materials and methods, Tables S11–S13). Prokaryotic alpha diversity calculated at genus level was significantly higher in BR2 compared to the BAY across all seasons (Supplementary material Fig. S8, *P* < .05). Alpha diversity across all samples was positively and negatively correlated with temperature (R = 0.62, *P* < .001) and DO (R = −0.52, *P* < .001), respectively (Fig. S8B; A and F). Beta diversity comparisons revealed that microbial communities clustered by sites and to a lesser degree by seasons (Fig. 3A; B), indicating the presence of unique spatial and temporal (summer and winter) niches. Spatial community distributions were linked to key environmental factors, for example, microbial compositions at site BR2 were mainly driven by nutrient availability (nitrate, silicon, and phosphorus; Fig. 3C). Temporal community profile comparisons revealed significant differences between summer and winter, in the observed time period (Fig. 3B), and identified temperature and DO as main temporal drivers (Fig. 3C), with further influences by turbidity and total dissolved solids.

### Prokaryotic taxa aligned with environmental factors indicate brackish and marine ecotypes

Prokaryotic communities across sites and seasons were mainly composed of *Bacteria* and to a lesser degree of *Archaea*, with average relative abundances of 73.63% and 26.36% (± 4.41 SD), respectively (Fig. S9A, Table S14). We detected 61 bacterial phyla, with the highest relative abundances for *Pseudomonadota* (Oren and Garrity 2021) [40] (homotypic synonym “Proteobacteria”), followed by *Actinobacteriota*, *Bacteroidota*, *Cyanobacteria*, *Marinisomatota*, and *Verrucomicrobiota* (Fig. 3D). Amongst the 18 archaeal phyla detected, *Thermoproteota* and *Thermoplasmatota* were most abundant (Fig. 3D). Community profiles at higher taxonomic resolution revealed that the 50 most abundant genera cumulatively accounted for an average of 78.81% (± 3.7%) of all taxa (Fig. S9B, Table S14), across sites and seasons. In total, 39 of the 50 genera were previously uncharacterised, i.e. they were only assigned to non-Latin placeholder names in the GTDB (Fig. S10).

We focused on these 50 most abundant genera and observed significant differences in abundance (*P* < .001) between BAY and BR2 (Fig. 3D, Table S15), as well as temporal trends, and correlations with environmental parameters (Fig. S11; A and B, Table S16). Pronounced spatial differences in relative abundances were detected between genera of multiple orders, including *Flavobacteriales*, *Pelagibacterales*, *Rhodobacterales*, *Pseudomonadales*, and SAR86. For example, four Flavobacteriales genera were significantly more abundant at the BAY site, and based on this observation we designated them as marine ecotypes (see Materials and methods). These genera included the newly named taxon *Eutrophosalina gen*. *nov*., which despite being present at all three sites with at least 17.8% average relative abundance in BR2 (Fig. 2), exhibited a strong negative correlation with nutrients, as well as a moderate positive correlation with salinity and pH (Fig. S11B). Contrarily, MED-G11, which occurred at higher abundances at the brackish site BR1, showed a moderate positive correlation with turbidity and nutrients (Fig. S11B). Within *Rhodobacterales*, the three genera, HIMB11, LGRT01, and *Salinivivens gen*. *nov*., had significantly higher abundances at the BAY (marine ecotypes), compared to the genus *Marivivens* that was significantly more abundant at BR2 (brackish ecotype; Fig. 3D). Abundances of the marine ecotype genera LGRT01 and HIMB11 were highly correlated with salinity and pH (Fig. S11B), whereas the brackish ecotype genus *Marivivens* showed strong negative correlation with salinity and DO (Fig. S11B). However, the ecotypes showed different temporal patterns, with LGRT01 abundances peaked in winter, whereas HIMB11, *Marivivens* and *Salinivivens gen. nov.,* showed the highest abundances in summer (Fig. 3D). As for the order Pelagibacterales, the two genera *Pelagibacter* and Pelagibacter_A had significantly higher abundances at the BAY, whereas four genera were enriched at BR2 (HIMB114, *Hypereutrophica gen*. *nov*., IMCC9063, and NG_56). All brackish ecotypes showed a strong positive correlation with nitrates and phosphorus (Fig. S11B), and some exhibited seasonal patterns, for example, IMCC9063 abundances peaked during winter, whereas novel group NG_56 abundances were highest during autumn.

Most genera of the order SAR86 had the highest relative abundances in the BAY, except for VMDH01, which showed higher abundances at BR2 (Fig. 3D). The marine ecotype genera (CAJQGY01, D2472, MED-G82, and SCGC-AA076-P13) had strong positive correlations with ammonia, whilst the brackish ecotype VMDH01 showed strong positive correlations with nitrate and TP (Fig. S11B). Amongst the order *Pseudomonadales*, three genera *Luminiphilus*, HTCC2207 and *Salivita gen*. *nov*. showed higher abundances in the BAY (marine ecotype), whereas the genus *Eutrophocola gen*. *nov*. was more abundant in BR2 (brackish ecotype). All three marine ecotype genera had moderate to strong negative correlation with nitrates and TP, whilst the brackish ecotype *Eutrophocola gen*. *nov*. showed a strong positive correlation with both nutrients (Fig. S11B, Fig. 3D). Additional members amongst the top 50 most abundant genera included Synechococcus_E (updated to *Parasynechococcus* in GTDB R214) from the order PCC-6307 (family *Cyanobiaceae*) which was highly abundant at the BAY site and peaked during spring, and the archaeal genus *Nitrosopumilus* (*Nitrosphaerales*) which was most abundant at BR2 and peaked during autumn (Fig. 3D).

### Metabolic profiling reveals distinct nutrient cycling capabilities of brackish and marine ecotypes

**Figure 4:**
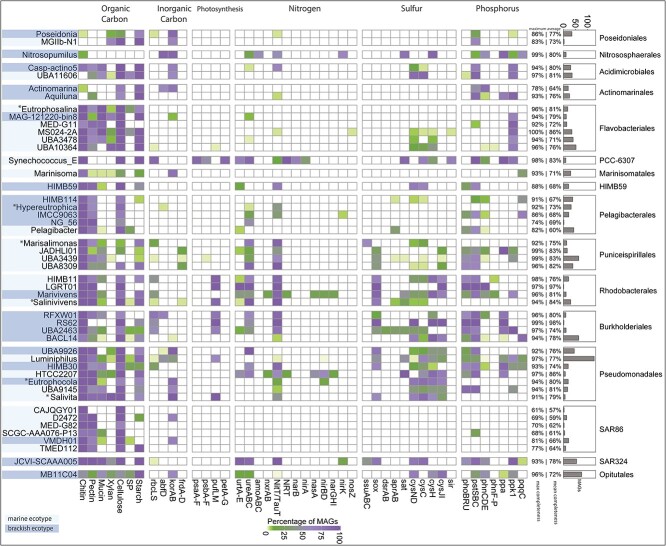
Metabolic profiles of the most abundant OTUs. The heatmap displays the metabolic profiles of the 50 most abundant genera by showing the percentage of MAGs (in rows) that carry key genes (in columns) involved in biogeochemical cycling, based on the KEGG/CAZY database. Note that a gene is only shown if it occurs in at least 10% of all MAGS in a given taxon. The genera designated as brackish (BR2) or marine (BAY) ecotypes are shaded in dark blue and light blue on the left (see legend). The percentage on the right indicates maximum and average completeness for all MAGs associated with a genus separated by a bar “|”. The bar plot represents the number of MAGs in each genus. The figure includes the newly proposed genus names *Eutrophosalina* (MED-G14), *Hypereutrophica* (SYDM01), *Marisalimonas* (AAA536-G10), Salinivivens (MED-G52), *Eutrophocola* (RGAU01), and *Salivita* (RFVC01) marked by asterisks (*).

An initial gene centric analysis, based on a 2.8 million nonredundant gene catalogue, revealed a diverse metabolic potential of the estuary microbiome covering most major nutrient cycling (carbon, nitrogen, phosphorus, and sulphur) pathways (Fig. S12, S13, Tables S17, S18). Subsequent metabolic profiling, focused on MAGs assigned to the 50 most abundant genera (Fig. 4; Table S19), enabled us to connect phylogeny and function, and to infer which taxa have the potential to drive biogeochemical cycles in the estuary (Table S20). Overall, all profiled abundant genera showed the metabolic capability to participate in organic carbon degradation (Fig. 4), e.g. they carried a wide range of genes for the degradation of complex polysaccharides such as pectin, chitin, cellulose, xylan, sulphated polysaccharides, arabinose, and starch (Fig. 4). The majority of genera were inferred to take part in phosphorus, sulphur, and nitrogen cycling, as expected of microbial taxa inhabiting a hypereutrophic estuary [48].

Diverse potential phosphorus acquisition strategies were observed in several taxa. For example, genes encoding high affinity inorganic phosphate (Pi) transporters (*pstSBC*) and in/organic phosphate metabolism and transport regulation (*phoBRU*) were present within multiple genera belonging to orders *Rhodobacterales*, *Burkholderiales*, and *Pseudomonadales* (Fig. 4). In addition, the *pqqC* gene, encoding for pyrroloquinoline quinine, a cofactor for several bacterial glucose/alcohol dehydrogenase [49] involved in solubilising inorganic phosphorus (iP), was present in the brackish *Pelagibacterales* genera, HIMB114, and IMCC9063. The *Rhodobacterales* genus *Marivivens* contained genes encoding for the utilisation of organophosphonate (*phnCDE* and *phnF-P*), an important component of dissolved organic phosphorus in the marine environment [50]. Inferred sulphur cycling pathways were prevalent in *Rhodobacteriales* and *Pseudomonadales*. Genera from both orders carried genes for thiosulphate oxidation (*sox*), and most *Pseudomonadales* and two *Rhodobacteriales* genera HIMB11, *Marinivivens* also encoded the entire assimilatory sulphate reduction pathway (Fig. 4). In addition, several MAGs assigned to the genera *Marisalimona*s *gen. nov.,* and UBA11606 from the order *Acidimicrobiales*, as well as order HIMB59 and genus JCVI-SCAAA005 from the order SAR324, carried genes inferred to reduce sulphate to sulphite (*cysNDC*).

Screening for nitrogen cycling capabilities recovered genera with the ability to degrade urea and to perform nitrate and nitrite reduction, as well as ammonia oxidation (Fig. 4). Urea degradation genes were prevalent in the abundant genera (Fig. 4), including *Parasynechococcus* (*Synechococcus_E*) and *Nitrosopumilus*. The latter genus was more abundant at the brackish BR2 site (Fig. 4), and possessed the capacity to oxidise ammonia catalysed by ammonia monooxygenase *(amoABC)*, and to reduce nitrite to nitric oxide (NO) by nitrite reductase (*nirK*). However, the gene for hydroxylamine dehydrogenase (*hao)*, which converts the amoABC product hydroxylamine to nitrite, could not be detected. [51]. The recently proposed species “*Candidatus* Nitrosopumilus aestuariumsis” from a subtropical estuary in southern China [13] prompted us to explore species level relationships. Based on the commonly accepted average nucleotide identity (ANI) threshold of 95%, all three species recovered in our study were distinct taxa compared to “*Ca*. N. aestuariumsis” (Table S21). To highlight the presence of novel *Nitrosopumilus* species in the Brisbane River estuary, we proposed the new taxon *Nitrosopumilus brisbanensis* (Table 1). Genes encoding nitrate reductase/nitrite oxidoreductase (nxrAB) were detected in Nitrospina which belongs to a group of nitrite-oxidising bacteria (NOB). *Parasynechococcus,* a marine ecotype, was inferred to use nitrogen for biosynthesis since this genus harbours genes for assimilatory nitrate reduction to ammonia (*narB* and *nirA*) (Fig. 4). Another genus with the potential to drive nitrogen cycling is *Marivivens* (*Rhodobacteraceae*), a brackish ecotype that encoded the complete pathway for dissimilatory nitrate reduction (DNRA) (*narGHI* and *nirBD*), and also nitrite oxidation (*nxrAB*).

*Parasynechococcus* was the only taxon that encoded an oxygenic photosynthetic system (*psa*, *psb*, and *pet*) (Fig. 4). Other taxa with photosynthetic capabilities were the *Rhodobacterales* genera HIMB11, LGRT01, and *Salinivens gen*. *nov*. (*Rhodobacterales*), the *Burkholderiales* genera RFXW01, RS62, UBA2463, and the *Pseudomonadales* genus *Luminiphilus* and *Salivita gen*. *nov*. (formerly RGAU01) of the order Pseudomonadales (Fig. 4). However, MAGs of these genera do not contain psa/psb/pet genes but possess *pufLM* genes, which are associated with aerobic anoxygenic photosynthesis [52] and carbon fixation via Calvin cycle (*rbcLS*), indicating autotrophic lifestyles. All of these genera encoded the genes for nitrate uptake (*NitT/TauT*) and the oxidation of thiosulphate (*sox*) to sulphate, which could function as electron donor for the anoxygenic photosynthesis (Fig. 4).

In addition to our inferred functional characterisation of dominant community members, we identified rare biosphere taxa, with a relative abundance of less than 0.1%, that have the potential to carry out important nutrient cycling (carbon, nitrogen, phosphorus, and sulphur) functions. For example, multiple genera of the order GCA002705445, and the genera VMDJ01 (order *Chromatiales*) and VMDI01 (order PS1) encoded genes for the dissimilatory sulphate reduction and oxidation pathway (*dsrAB*, *aprAB*, and *sat*) (Fig. S14). Genes for nitrate reduction to ammonia (DNRA; *narGHI/napAB* and *nirBD*) and denitrification (*nirS*, *norBC*, and *nosZ*) were present in the genus *Thalassospira* (order Rhodospirillales). Several genera in the order *Nitrospinales* and the genus *Alcanivorax* (*Pseudomonadales*) also encoded genes involved in nitrite reduction to ammonia (*nirA* and *nrfAH*) or to NO (*nirK*), implying a role in estuarine nitrogen cycling. In addition, genus *Alcanivorax* possessed the key gene (*abfD*) for the 3-hydroxypropionate/4-hydroxybutyrate cycle, a carbon fixation pathway originally described in *Archaea* but recently also discovered in Alpha- and Gammaproteobacteria [53]. Furthermore, the newly proposed genus *Eutrophovita gen*. *nov*. encoded a partial DNRA pathway and nitrate transporters (*NitT/TauT* and *nirBD*) next to a complete assimilatory sulphate reduction pathway. The methane-related pmoABC cluster was only identified in the genus *Cycloclasticus* (order *Methylococcales*), which contains aromatic hydrocarbon degraders [53] (Fig. S14B). Genomes of the recently proposed genus *Pseudoprimorskyibacter* (order *Rhodobacterales*) encoded anoxygenic photosynthesis (*puflM*) and a pathway for nitrate transport (*NitT/TauT*) and the oxidation of thiosulphate (sox) to sulphate. A wide range of other rare biosphere taxa, including genera of the orders *Vicinamibacterales*, *Rhodobacterales*, *Rhodospirillales*, and *Pseudomondales*, also carried the gene for polyphosphate accumulation (*ppk1*) and could represent putative polyphosphate accumulating organisms (PAOs) [54]. Patescibateria might also contribute to nutrient cycling at site BR2, since genera of this phylum carried genes for chitin degradation, and encoded nitrite reductase (*nirK*), sulphate adenylyltransferase (sat), and polyphosphate kinase (*ppa*) (Fig. S14B). Based on their reduced estimated genome sizes, ranging from 1 to 1.8 Mbp (Table S4), these taxa might be symbionts or even parasites, similar to the nonphotosynthetic cyanobacterial genus CAIYXB01 (class *Vampirovibriona*), which was more abundant at BR2, representing a brackish ecotype.

### Machine learning-based automated recovery identifies environmental indicator taxa

**Figure 5:**
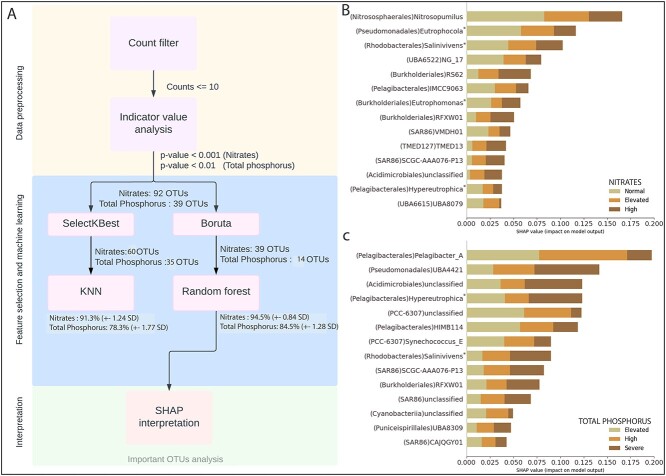
Machine learning (ML) workflow and SHapely Additive exPlanations (SHAP) plots. A) ML workflow depicting the three main steps in processing taxa count data (relative OTU abundances): (i) selecting features, (ii) building models, and (iii) interpreting the results. SHAP interpretation (mean SHAP values) for B) nitrates and total C) phosphorus, respectively. Shown are the top OTUs based on the random Forest model, whereby the OTUs are ranked by their impact on the model’s prediction, using the mean absolute SHAP value for each nutrient class of nitrate and TP. For example, *Nitrosopumilus* has the highest impact on the nitrate model overall, and the Pelagibacter_A has the highest impact on the TP model.

To identify microbial genera with ecological relevance, we focused on nitrate and TP, since the levels of both nutrients were strongly elevated in the hypereutrophic Brisbane River estuary (Fig. S1). Our workflow consisted of three main blocks: (i) data preprocessing, including an indicator value analyses (IVA) to eliminate redundant OTUs, followed by (ii) feature selection and machine learning (ML), and (iii) model interpretation (Fig. 5A). The data preprocessing part of the workflow included an initial prefiltering step, after which 1107 OTUs remained. Next the IVA identified 92 and 39 OTUs as significant indicators for nitrate (*P* < .001) (Table S22) and TP (*P* < .05), respectively (Table S23). In the feature selection and ML step, we trained two different classifiers, KNN and Random Forest, on the features selected by SelectKBest and Boruta, respectively (Fig. 5A). The KNN classifier was trained on a SelectKBest feature selection of 60 OTUs for nitrates and 35 OTUs for TP. In contrast, the Random Forest classifier was trained on a subset of 39 OTUs for nitrate and 14 OTUs for TP selected by Boruta (Fig. 5A).

We evaluated ML models on the full OTU dataset, and the resulting cross-validation accuracy for KNN classifier was 91.3% (± 1.24 SD) for nitrates and 78.3% (±1.77 SD) for TP, whereas the cross-validation accuracy for the Random Forest classifier was 94.5% (± 0.84 SD) for nitrates and 84.5% (± 1.28 SD) for TP (Fig. 5A). Since cross validation accuracy can be used as approximations for predictive model performance, we picked the better performing classifier, i.e. Random Forest, for the downstream SHapely Additive exPlanations (SHAP) interpretation of ML models. SHAP deconstructs each prediction by quantifying the impact (SHAP values) of every input feature (OTU) on the ML model. We selected the top fourteen OTUs that had pronounced differences amongst the classes for nitrates and TP (Fig. 5A). These OTUs (features) were then ranked according to their impact on the classification model, based on the mean absolute SHAP value across all 54 samples (Fig. 5; B and C). Thereby, SHAP values are shown for each nutrient class, normal to high for nitrate, and elevated to severe for TP (see Materials and methods; Table S22 and S23). Mean absolute SHAP values identified genera from several orders including *Pelagibacterales*, *Nitrososphaerales*, *Rhodobacterales*, *Pseudomonadales*, *Burkholderiales*, SAR86, and PCC-6307 (Fig. 5B and C, S13). Several newly named taxa were also identified as important indicators, such as *Eutrophocola gen*. *nov*. and NG_17 (order UBA6615), and *Eutrophomonas gen*. *nov*. for nitrates, and the genera *Hypereutrophica gen. nov.,* (formerly SYDM01, order *Pelagibacterales*) and Salinivivens *gen*. *nov*. (*Rhodobacterales*) for both nitrates and TP (Fig. 5B and C).

**Figure 6:**
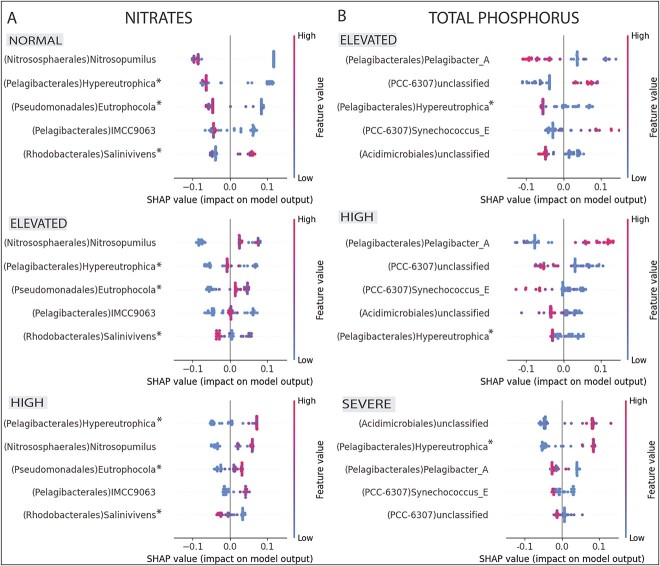
ML approach to predict indicator taxa for nitrate and TP concentrations. Both A) and B) show bee swarm plots for nitrate and TP, respectively, focusing on the top five OTUs and their positive and negative impact on the model prediction. The feature value is indicated by colour gradients. For example, higher abundances (red colour) of *Salinivivens* have a positive impact on the normal nitrate conditions (class “normal”).

The SHAP analyses revealed several key OTUs (genera) with pronounced differences for some nitrate and TP classes. For example, the lower abundances of the genera IMCC9063 (*Pelagibacterales*), *Nitrosopumilus* (*Nitrososphaerales*) and Eutrophocola *gen*. *nov*. (*Pseudomonadales*) had a positive impact on the “normal” nitrate class, and higher abundances had a positive impact on the “high” nitrate class (Fig. 6). We observed the opposite trend for genus *Salinivivensgen*. *nov*. (formerly MED-G52, *Rhodobacterales*), i.e. higher abundances had a positive impact on the “normal” class, and lower abundances had a positive impact on the “high” nitrate class (Fig. 6). As for TP, higher abundances of genus *Parasynechoccocus* had a positive impact on the model classification of the “elevated” class, whereas an unclassified genus of the order *Acidimicrobiales*, had a positive impact on model prediction in the “severe” class (Fig. 6). The only taxon identified in both high nutrient predictions was *Hypereutrophicagen*. *nov*. (formerly SYDM01, order *Pelagibacterales*) with high abundances having a positive impact on model prediction for the “high” nitrate and “severe” TP classes, which makes this genus an important indicator for both nutrient categories (Fig. 6), hence the name.

## Discussion

Our results revealed that the Brisbane River estuary represents a hypereutrophic ecosystem harbouring a diverse but mostly uncharacterised prokaryotic community. We implemented a spatio-temporal metagenomics and ML approach to assess diversity and distribution of estuarine communities, to identify bacterial and archaeal key taxa, and to infer their ecosystem functions.

### An anthropogenically stressed ecosystem shaped by excessive nutrient loads

The elevated total nitrogen (TN) and TP concentrations recorded during our 1.5 year sampling period (Fig. S1, Table S1) are supported by previous reports of high nutrient loads [55] and suggest that the Brisbane River estuary is an ideal model system to study hypereutrophic ecosystems caused by anthropogenic activities. Agricultural runoff, aggravated by extensive fertiliser use, and wastewater treatment plant (WWTP) effluents, account for 40 to 70% of TN and TP in the Brisbane River estuary [56, 57]. We found that TN values, in the form of ammonium (NH_4_^+^), nitrate (NO_3_^+^) and nitrite (NO_3_^2−^), and TP inputs, mainly as inorganic phosphate (PO_4_^3−^), were 5–20× higher than the water quality thresholds issued by the Queensland government [46], suggesting that efforts to reduce TN and TP levels in the Brisbane River over the past decades had limited success [58, 59]. As expected, nitrate and phosphorus were amongst the main drivers of the microbial community at the brackish site BR2, which experiences the highest levels of agricultural runoff and WWTP discharge [58]. We also recorded the lowest DO values at BR2, especially in summer when DO averaged only 5.49 ± 0.36 mg O_2_/l (Table S1), which is below the local estuarine threshold of 7–9 mg O_2_/l [46] suggesting that eutrophication decreases oxygen availability [60] at this location.

### Nitrate and phosphorus cycling inferences highlight the roles of uncharacterised and novel taxa

**Figure 7:**
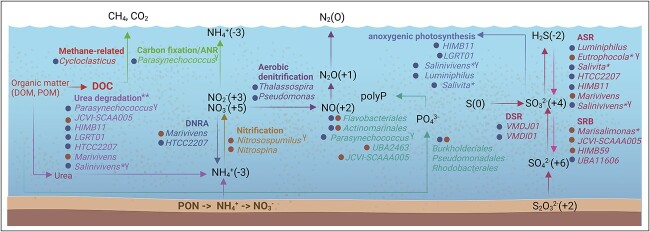
Conceptual diagram of biogeochemical functions taking place in Brisbane river. Main pathways involved in nutrient processing (carbon, nitrogen, phosphorus, and sulphur) are shown. Substrates are presented with valency in brackets to indicate oxidation/reduction processes. Genera inferred to be involved in nutrient cycling are marked as marine or brackish ecotypes (based on MAG abundance) with blue or brown circles, respectively. Indicator taxa, derived by ML, are indicated by a “ɣ” symbol. A single asterisk (*) indicates newly named taxa. Double asterisks (**) indicate that only the most abundant urea degrading genera are shown, for a full list see Table S20. Chemical formulas include: CH_4_ = methane, CO_2_ = carbon dioxide, NH_4_ = ammonium, NO_3_(=%) nitrate, NO_2_ = nitrite, NO = nitric oxide, N_2_O = nitrous oxide, N_2_ = dinitrogen, PO_4_ = phosphate, S_2_O_3_ = Thiosulphate, SO_4_ = sulphate, SO_3_ = sulphite, H_2_S = hydrogen sulphide, and S = elemental sulphur. Acronyms: Particulate organic nitrogen (PON), polyphosphate (polyP), dissimilatory nitrate reduction (DNRA), assimilatory sulphate reduction (ASR), dissimilatory sulphate reduction and oxidation (DSR), sulphate-reducing bacteria (SRB) Created with Biorender (TM)

Our results revealed that many essential nutrient cycling (carbon, nitrogen, phosphorus, and sulphur) functions, which have the potential to influence ecosystem dynamics, are encoded by previously uncharacterised taxa, in particular at the genus level (Fig. 6 and 7). This finding supports the conclusion that microbial taxa in subtropical, hypereutrophic estuaries are vastly underexplored. Our inferred metabolic pathways and enzymes linked to bacterial and archaeal genomes are a first, pivotal step to understand the role of these microbes in maintaining ecosystem functions.

In regard to nitrogen cycling, inferred nitrogen conversion and removal processes, including DNRA, aerobic denitrification, and nitrification [20, 61] were present throughout the estuary but showed higher abundances at BR2, the site with the highest nitrogen concentrations (Fig. S1). Although DNRA has been mainly reported from anaerobic habitats [62–64], this pathway could also function in oxic environments [20]. DNRA encoding taxa identified in our study include the marine ecotype HTCC2207 and the brackish ecotypes *Marivivens* (*Pseudomonadales*) and the proposed genus Eutrophocola *gen. nov*., the latter of which encoded only a partial DNRA pathway. These three genera likely compete with aerobic denitrifiers, which simultaneously utilise both oxygen and nitrate as electron acceptors [65], to retain nitrogen in its most bioavailable form as ammonium [66]. However, the entire denitrification pathway was only encoded by the rare taxa (< 0.1% relative abundances) *Thalassospira* and *Pseudomonas* (Fig. S14), indicating that aerobic denitrification is not the dominant nitrate removal pathway in the sampled estuarine surface waters.

Nitrification appears to be primarily carried out by ammonia oxidising archaea (AOS) and nitrite-oxidising bacteria (NOB) in the estuary. In our dataset, the AOA genus *Nitrosopumilus*, characterised as a brackish ecotype (BR2, Fig. 6), was identified as an important indicator taxon for the highest concentration of nitrates by random forest ML. MAGs from this genus, including the proposed species *Nitrosopumilus brisbanensis*, encoded genes for urea degradation and ammonia oxidation (Fig. S14), a trait they have in common with the recently proposed species “*Candidatus* Nitrosopumilus aestuariumsis” from a subtropical estuary in China (Zou et al. 2020) [13]. All *Nitrosopumilus* genomes lacked a hydroxylamine dehydrogenase gene, which has been reported to be absent in this taxon [51], but encoded the oxygen-tolerating nitrite reductase (nirK). This latter enzyme could be used to reduce the toxicity of accumulated nitrite within cells [67] by converting it to NO. Conversion of ammonia to nitrite and subsequently to NO and even nitrous oxide, which has been proposed previously [68, 69], could explain the low abundances of NOB in the estuary. NOB identified in our dataset include the lesser abundant genera *Nitrospina* and UBA8687 from the order *Nitrospinales* which carried nitrite oxidoreductase (*nxrAB*) and nitrite reductase (*nirK*) genes. *Nitrospina* is also commonly present in wastewater treatment plants [70–72], indicating the preference of this genus for hypereutrophic habitats.

Amongst the ML defined indicators for the highest nitrate class were two *Pelagibacteriales* genera, including the highly abundant genus IMCC9063 and the so far unexplored genus *Hypereutrophica gen*. *nov*. (formerly SYDM01), both of which we identified as brackish ecotypes (Fig. 4). These results support previous studies reporting that *Pelagibacteriales* (synonym “SAR11 Clade”) contains several marine and brackish ecotypes [73]. The association of IMCC9063 and *Hypereutrophica gen*. *nov*. with high nitrate levels could be explained by the presence of nitrate ABC transporters (*Nit/TauT*), and urea breakdown genes (*ureABC*) to produce ammonia, both of which has been suggested for IMCC9063 previously [73].

Taxa with the potential to drive phosphorus cycling include *Parasynechococcus*, a marine ecotype (Fig. 3), that was identified as an important indicator for “elevated” levels of TP by the random forest models (Fig. 7). MAGs assigned to this genus carried multiple phosphorus solubilization genes (*pstSBC*, *phnCDE*, and *ppa*), suggesting that *Parasynechococcus* could be an important player in phosphorus cycling at the BAY site of the estuary. In addition, our data also indicates the widespread capacity for (in)organic phosphate transport and mineralisation (pstSBC, phoBRU, and phnCDE) and polyphosphate synthesis (*ppk1*) (Fig. 7), with the most complete set of genes detected in the genera belonging to orders *Puniceispirillales*, *Rhodobacterales*, *Burkholderiales*, and *Pseudomonadales*. It is tempting to speculate that some of these taxa could be particle associated, especially genera *Pelagibacter* and IMCC9063, both of which encode the alkaline phosphatase (APA) genes (*phoA* and *phoB*). APA mineralizes organic phosphorus into PO_4_ and high APA potential has been attributed mainly to particle attached bacteria [74, 75]. Another use of organophosphorus compounds has previously been proposed in the aerobic methane production hypothesis. Thereby, organisms use methylphosphonic acid as a resource for phosphorus and produce methane as a byproduct that is likely responsible for methane supersaturation in surface waters [76]. The reaction requires the carbon-phosphorus enzyme lyase complex which is composed of phosphonic acid transport (*phnCDE*) and phosphonate utilisation (*phnGHIJKLM*). We detected the complete set of genes for this complex in the highly abundant brackish ecotype genus *Marivivens* (*Rhodobacterales*) and the less abundant marine ecotypes *Pseudoprimorskybacter* and *Thalassospira* (*Rhodospirillales*).

### Carbon cycling is influenced by a turbidity driven shift from autotrophy to heterotrophy

Another anthropogenic impact on riverine ecosystems, including the Brisbane River estuary, is increased turbidity (Fig. S1). High turbidity levels reduce light penetration in surface waters and limit photosynthesis, thereby influencing microbial community compositions [77]. The lowest turbidity in our dataset was recorded at the BAY site, especially in winter (Fig. S1), resulting in higher light penetration in surface waters [77, 78]. This trend was supported by a differentially higher abundance of photosynthesis genes (*psa, psb*) in the BAY (Fig. 4), which were exclusively carried by the genus *Parasynechoccus* (Synechoccocus_E). However, we found evidence that aerobic anoxygenic phototrophs (AAP) might also be involved in the carbon cycling at the BAY site. AAPs are known to harness light energy to accumulate organic carbon, which would otherwise be respired, and use it for growth [79]. Genes for aerobic anoxygenic photosynthesis (*pufLM*) were present in a variety of genera, including LGRT01, HIMB11 and *Salinivivens* gen nov. (formerly MED-G52, order *Rhodobacterales*), as well as the genus *Salivita* gen nov. (formerly RGAU01, order *Pseudomonadales*), all of which were found to carry *pufLM* genes and were more abundant in the BAY. A dependency on anoxygenic photosynthesis could explain why *Salinivivens gen nov.,* was identified as indicator species for the lowest nitrate concentrations in our ML approach, given that nitrate concentrations were directly correlated with turbidity (Fig. S1) and higher turbidity implies limited light availability. This observation also highlights the fact that defining indicator taxa of nutrient pollution, based on metadata correlated abundances, needs to consider the metabolic potential of the taxa in question to determine if they are responding directly or indirectly to the tested nutrient conditions. All aforementioned AAP taxa also carried and expressed the sox gene operon for thiosulphate oxidation providing evidence that these taxa use sulphur as an electron donor for photosynthesis, as previously suggested for a marine *Roseobacter* clade [80].

The significantly higher turbidity at site BR2 correlated with significantly lower abundances of photosynthesis genes compared to all other sites (Fig. S3) and signalled a shift from an autotrophic to a heterotrophic ecosystem upstream. This trend was enhanced by temporal chemico-physical patterns and reflected in microbial abundance changes. For example, the *Flavobacteriaceae* genus MED-G11, showed an overall moderate positive correlation with turbidity and an abundance peak in spring (Fig. 3), a season where mudbank erosion causes increased turbidity in the estuary [81]. Thereby MED-G11, a motile heterotrophic degrader of chitin, pectin, mucin, and cellulose, could benefit from plant debris derived polysaccharides that have accumulated in the deposited mud over the winter [82, 83].

## Conclusion and outlook

Our study resembles the first attempt to link the ecological status of a hypereutrophic, subtropical estuary to high resolution microbial community profiles derived from metagenomic data. Developing a habitat specific genome reference database, resulted in an improved taxonomic coverage and allowed us to predict brackish and marine ecotypes. We detected high abundances of uncharacterised and novel taxa, particularly at the genus level, inferred their metabolic traits and described 11 new genera. Our results highlight the need for aquatic microbial ecology studies to move beyond community profiles that solely report higher taxonomic levels, i.e. phylum to family ranks, and to embrace genome-based methods that facilitate improved taxonomic resolution. We envision that the approach presented in this study will provide a blueprint for future analysis, and that creating sample specific genome reference databases prior to community profiling will soon become the standard in the field.

The combination of ML and metabolic reconstruction allowed us to identify ecologically relevant genera as microbial indicator taxa for nitrate and phosphorus concentrations. Thereby, our study built upon previous approaches of threshold indicator taxa derived from datasets that relied solely on chemico-physical metadata correlated with microbial abundances, e.g. amplicon sequence variants based metabarcoding. Whilst these methods have provided valuable primary data for biomonitoring [84–86], our metagenomic approach, combined with ML, has allowed us to gain insights into the metabolic potential of bacterial and archaeal indicators in an effort to understand the functional framework of the observed distributions. In particular, metagenomic data enabled us to infer if these taxa are responding directly or indirectly to the different categories of nutrient concentrations. Recently, comprehensive studies have explored the microbial communities of another subtropical estuary, the hypereutrophic Pearl River Delta in China [11, 12], and have provided insights into the spatio-temporal patterns of microbial diversity and functional potential. We envision that this rapidly growing dataset will enable further ML explorations of microbial links to river ecology in the near future.

## Supplementary Material

Combined_supplementaryfigures_251023_edited_ycae067

Supplementary_tables_May2024_ycae067

Supplementary_Text_June2023_docx_ycae067

## Data Availability

The reads analysed during the current study are available in the NCBI SRA repository (SRR26337510-78) and the MAGs have been deposited in BioProject PRJNA1024631 The R script for the sequence analyses is available on Github (https://github.com/aprabhu90/Brisbane-river-microbiome). The Python script for the ML analyses is available on Github (https://github.com/santule/microbe-ind).
