# Supplementary material for: Machine learning and metagenomics identifies uncharacterized taxa inferred to drive biogeochemical cycles in a subtropical hypereutrophic estuary
Source: ISME Commun. 2024 May 10;4(1):ycae067. doi: 10.1093/ismeco/ycae067 (PMC11758582; doi:10.1093/ismeco/ycae067)
Supplement: Combined_supplementaryfigures_251023_edited_ycae067 [file combined_supplementaryfigures_251023_edited_ycae067.pdf]

FIGURE S1

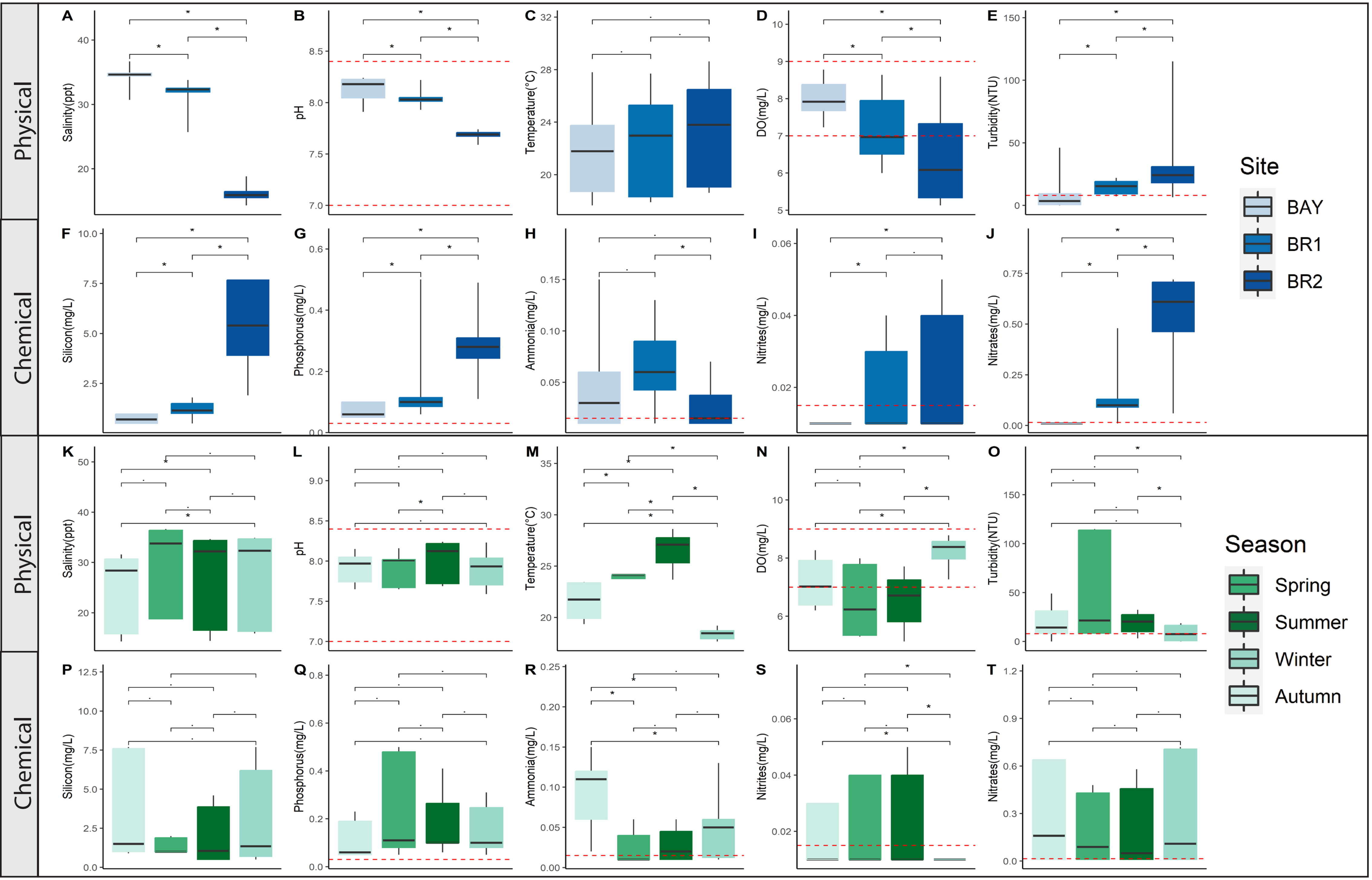

Figure S1 | Variation of the physical and chemical parameters measured across sampling sites and season. Shown are data across sampling sites (A-J) and seasons (K-T). Where available, threshold values, issued by Dept of Environment and Resource Management for the Brisbane river estuary, are indicated with red dashed lines. Pairwise comparisons were calculated using Wilcoxon's rank sum test to analyze significant changes across seasons and sites (. - $p< 0.05$,* - $p<0.01$) followed by Kruskal-Wallis post-hoc analyses. Acronyms are the brackish Brisbane River estuary (sites "BR1" & "BR2") and Moreton Bay (site "BAY").

FIGURE S2

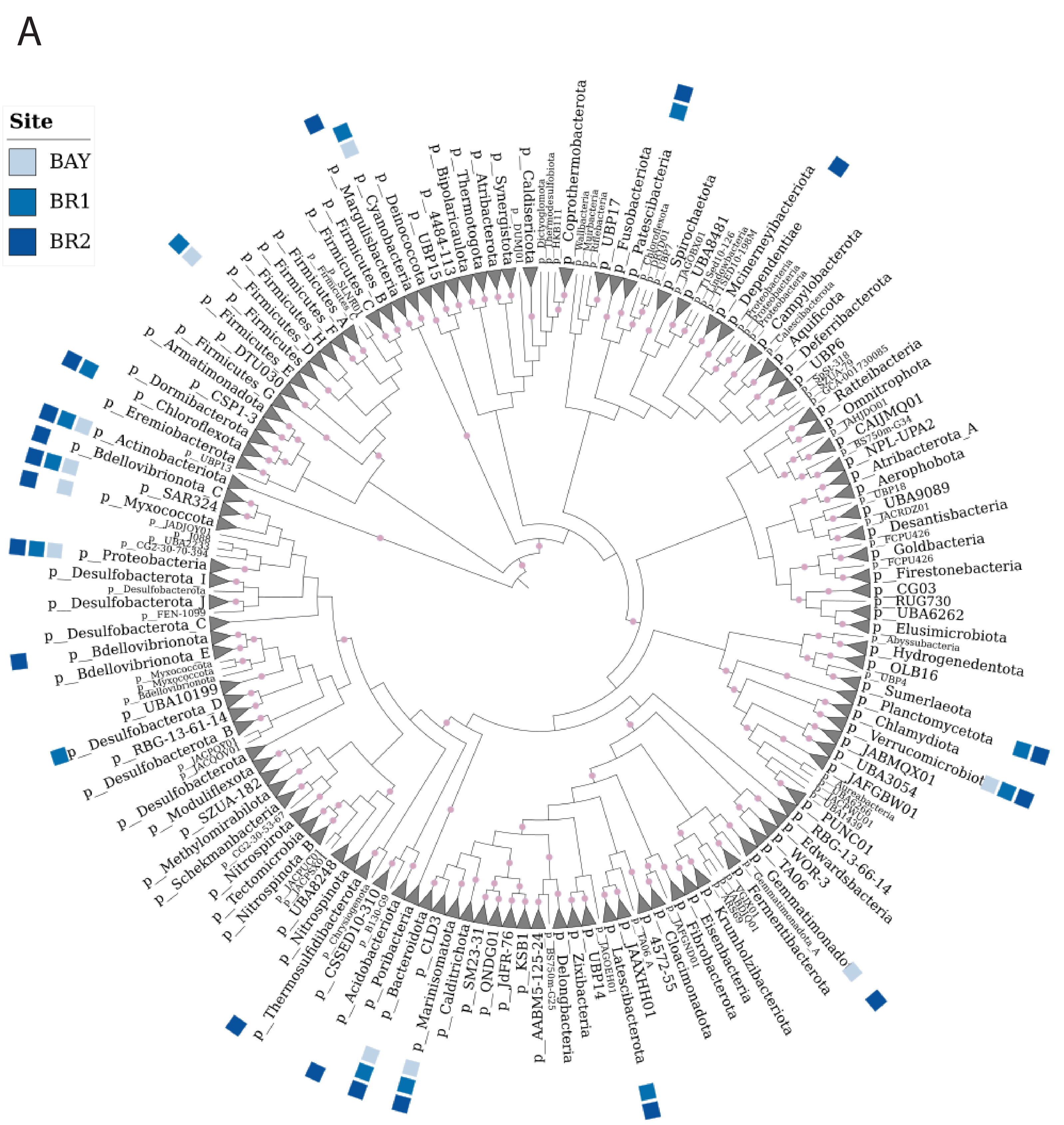

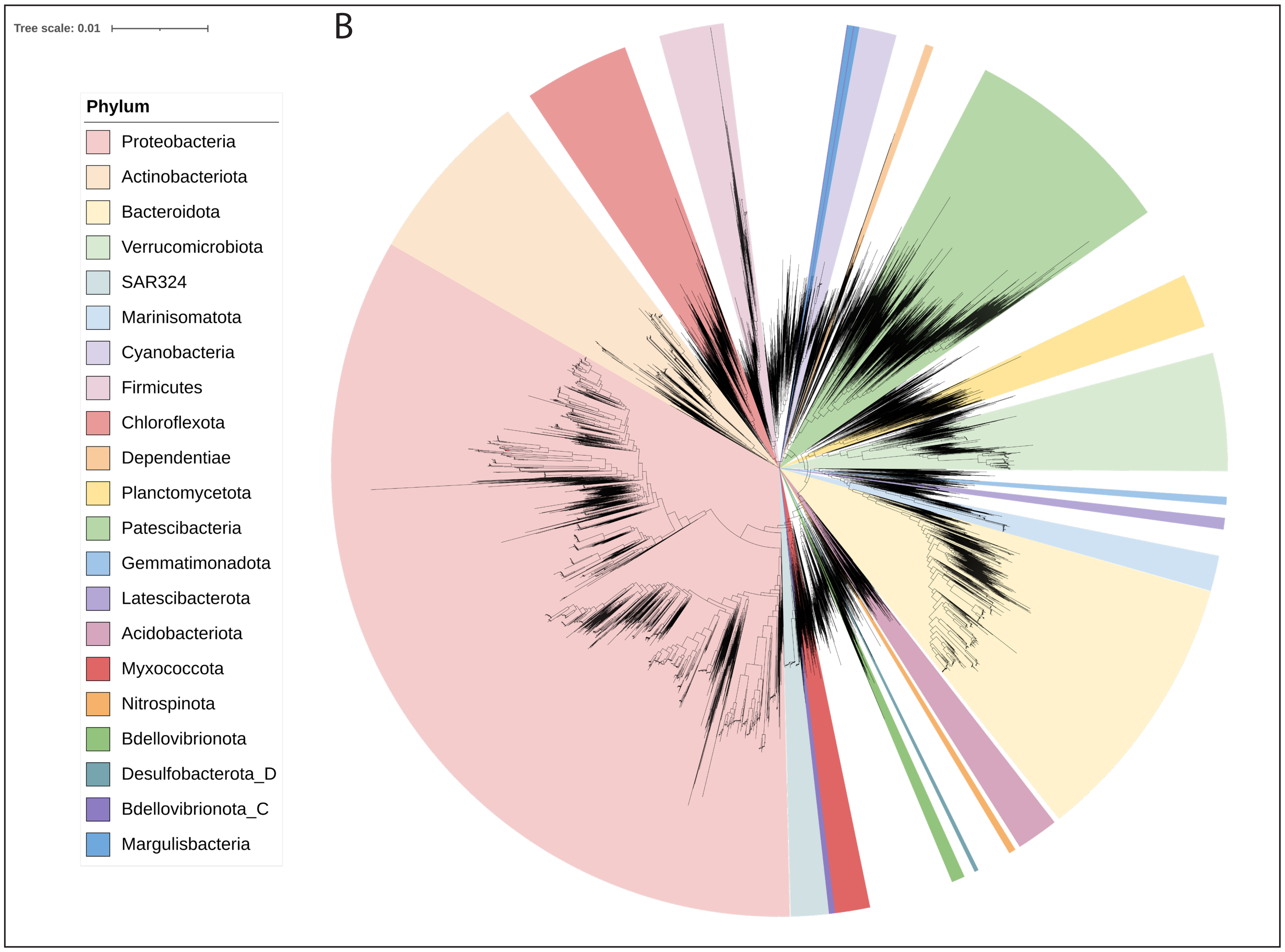

Figure S2 Phylogeny of bacterial MAGs recovered in the study. A) Bacterial phylogenetic tree (FastTree2) indicating distribution of MAGs recovered from the study, from sampling locations as shown in blue shades B) Bacterial phylogenetic tree (FastTree2) displaying distribution of MAGs, shaded by Phylum,as shown in the legend.

FIGURE S3

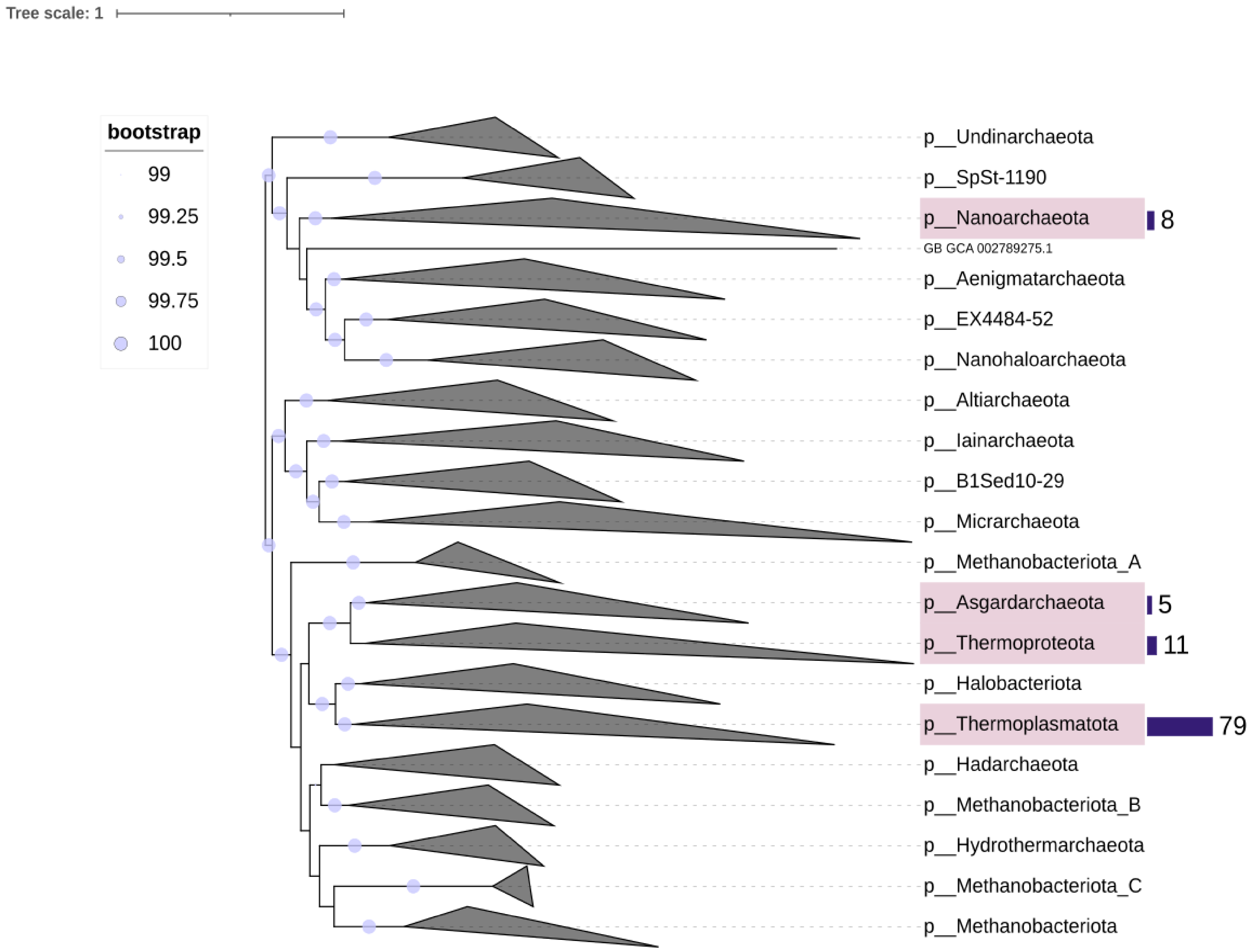

Figure S3 Phylogeny of archaeal MAGs recovered in the study. Bootstrapped bacterial phylogenetic trees (shown >= 99%) collapsed at phylum level along with MAGs recovered from each site. The sampling site is indicated by coloured squares, and the number of MAGs recovered from each lineage is provided in brackets.

FIGURE S4

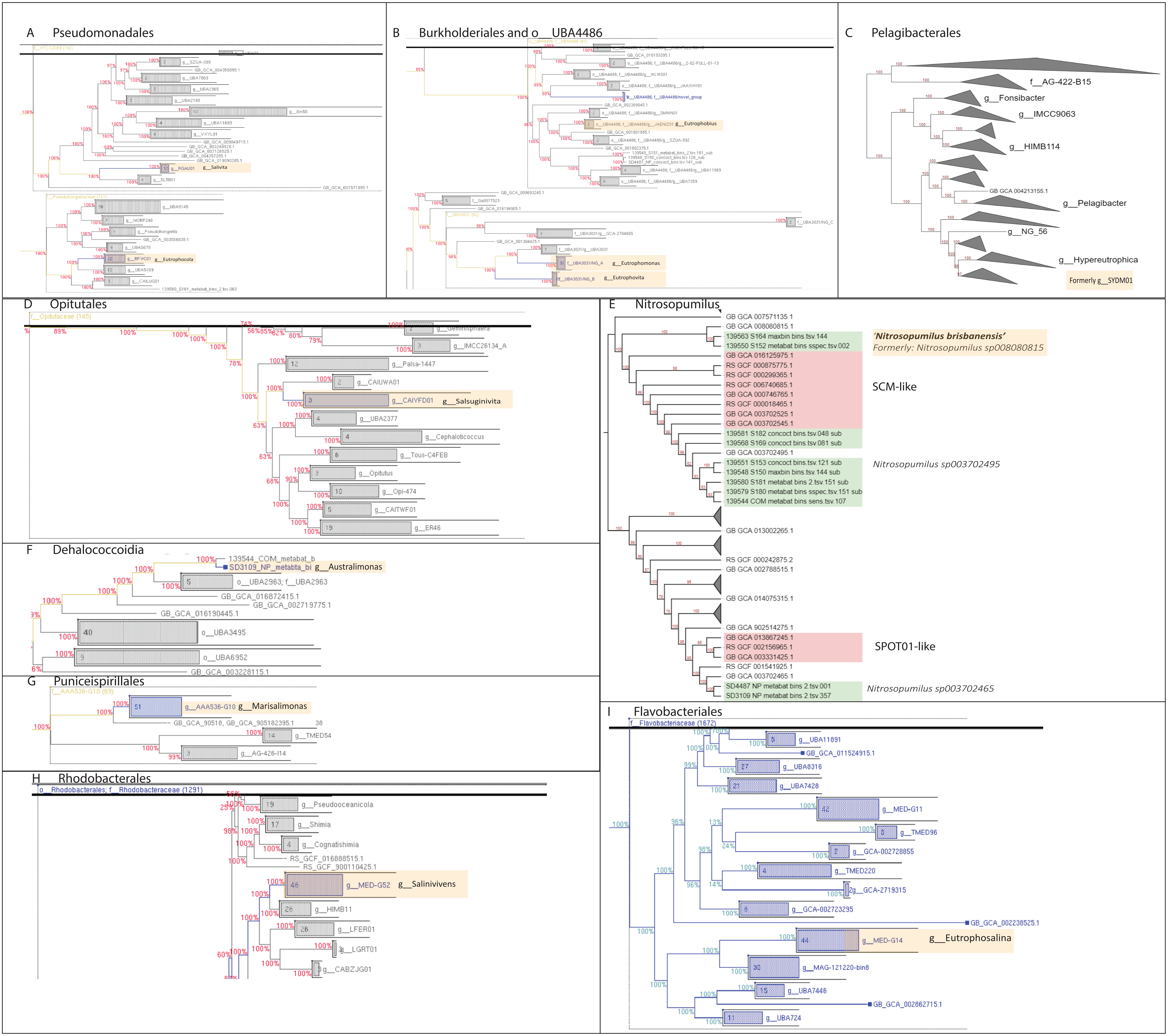

Figure S4 Phylogeny of novel and uncharacterised genera in the study. Bootstrapped (>=90) bacterial and archaeal phylogenetic trees for selected novel and uncharacterised genera, after rank normalisation based on relative evolutionary divergence (RED), collapsed at genus level. All genera appear to be on stable phylogenetic nodes (>99%) and belong to different ranks; A) Pseudomonadales tree B) Burkholderiales and o__UBA4486 C)Pelagibacterales D) Opitutales E)Nitrosopumilus F) Dehalococcoidia G)Puniceispirillales H) Rhodobacterales and I)Flavobacteriales

FIGURE S5

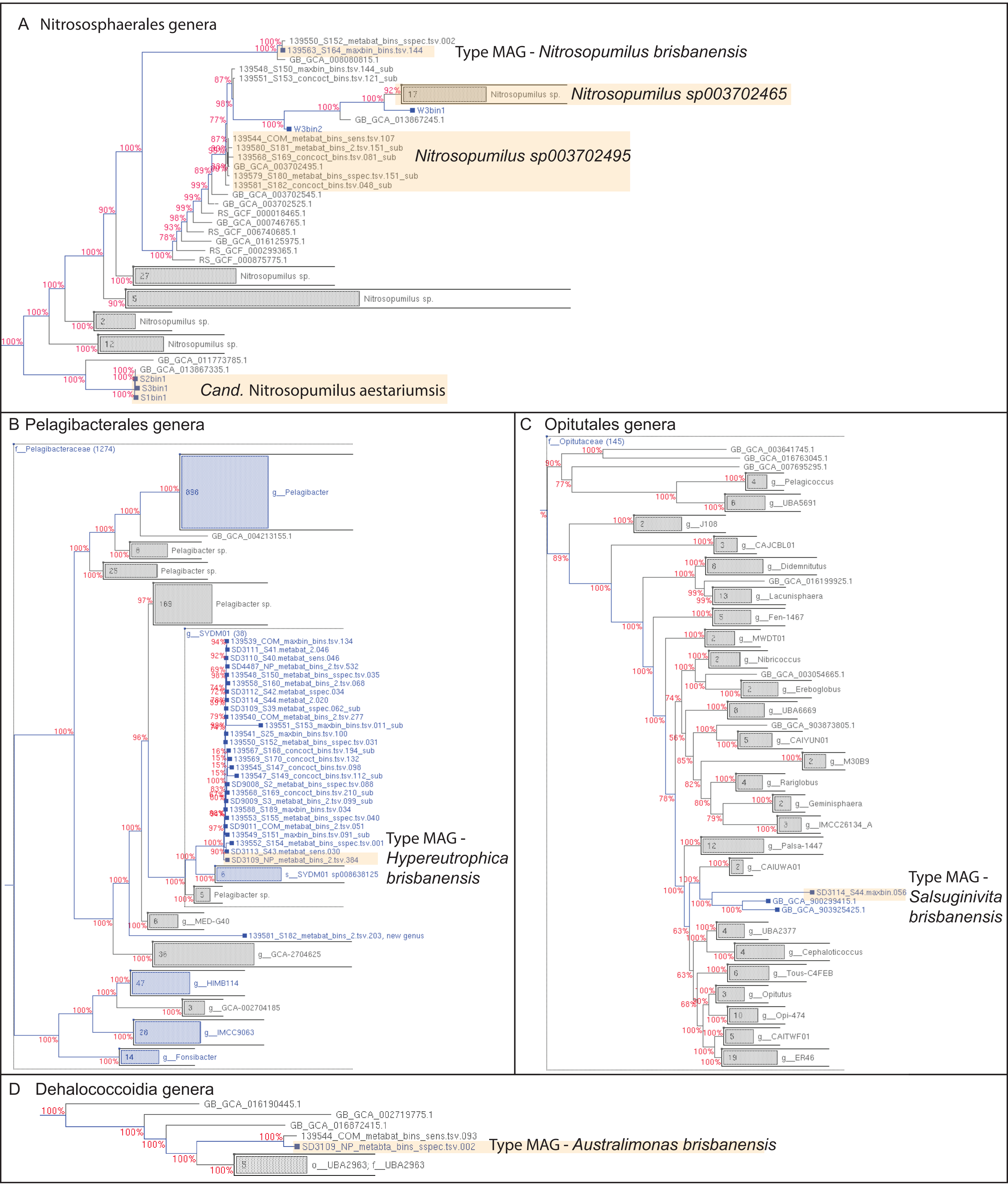

Figure S5 Phylogeny of novel and uncharacterised genera in the study. Bootstrapped (>=90) bacterial and archaeal phylogenetic trees for selected novel and uncharacterised genera, after rank normalisation based on relative evolutionary divergence (RED). All genera appear to be on stable phylogenetic nodes (>99%) and belong to different ranks, and the type MAG for each newly named genus is indicated; A) Nitrososphaerales B)Pelagibacterales C) Opitutales D) Dehalococcoidia

FIGURE S6

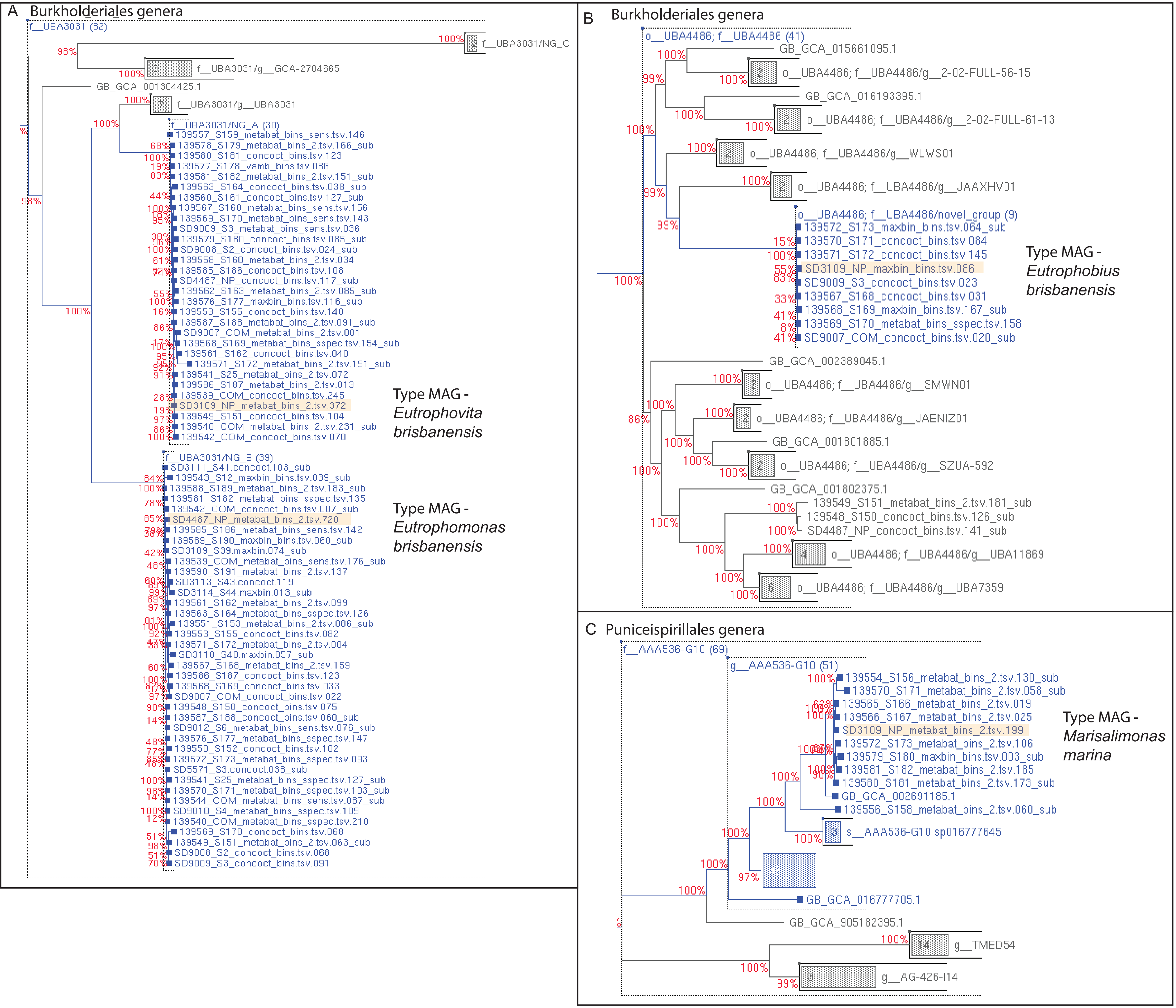

Figure S6 Phylogeny of novel and uncharacterised genera in the study. Bootstrapped (>=90) bacterial phylogeny for selected novel and uncharacterised genera, after rank normalisation based on relative evolutionary divergence (RED). All genera appear to be on stable phylogenetic nodes (>99%) and belong to different ranks, and the type MAG for each newly named genus is indicated; A) and B) Burkhoderiales genera C) Puniceispirillales

FIGURE S7

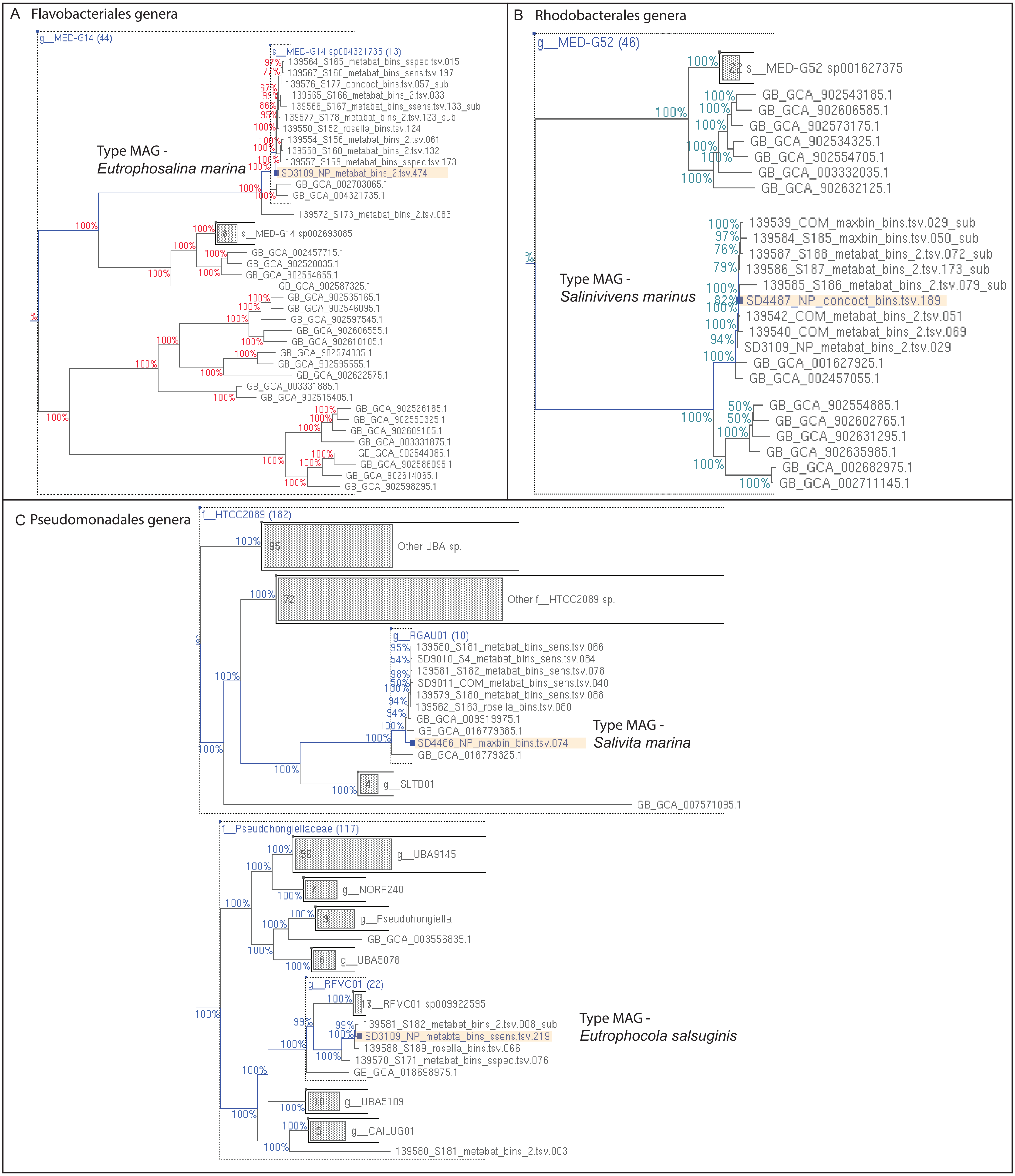

Figure S7 Phylogeny of novel and uncharacterised genera in the study. Bootstrapped (>=90) bacterial phylogeny for selected novel and uncharacterised genera, after rank normalisation based on relative evolutionary divergence (RED). All genera appear to be on stable phylogenetic nodes (>99%) and belong to different ranks, and the type MAG for each newly named genus is indicated; A) Flavobacteriales B) Rhodobacterales C) Pseudomonadales

FIGURE S8

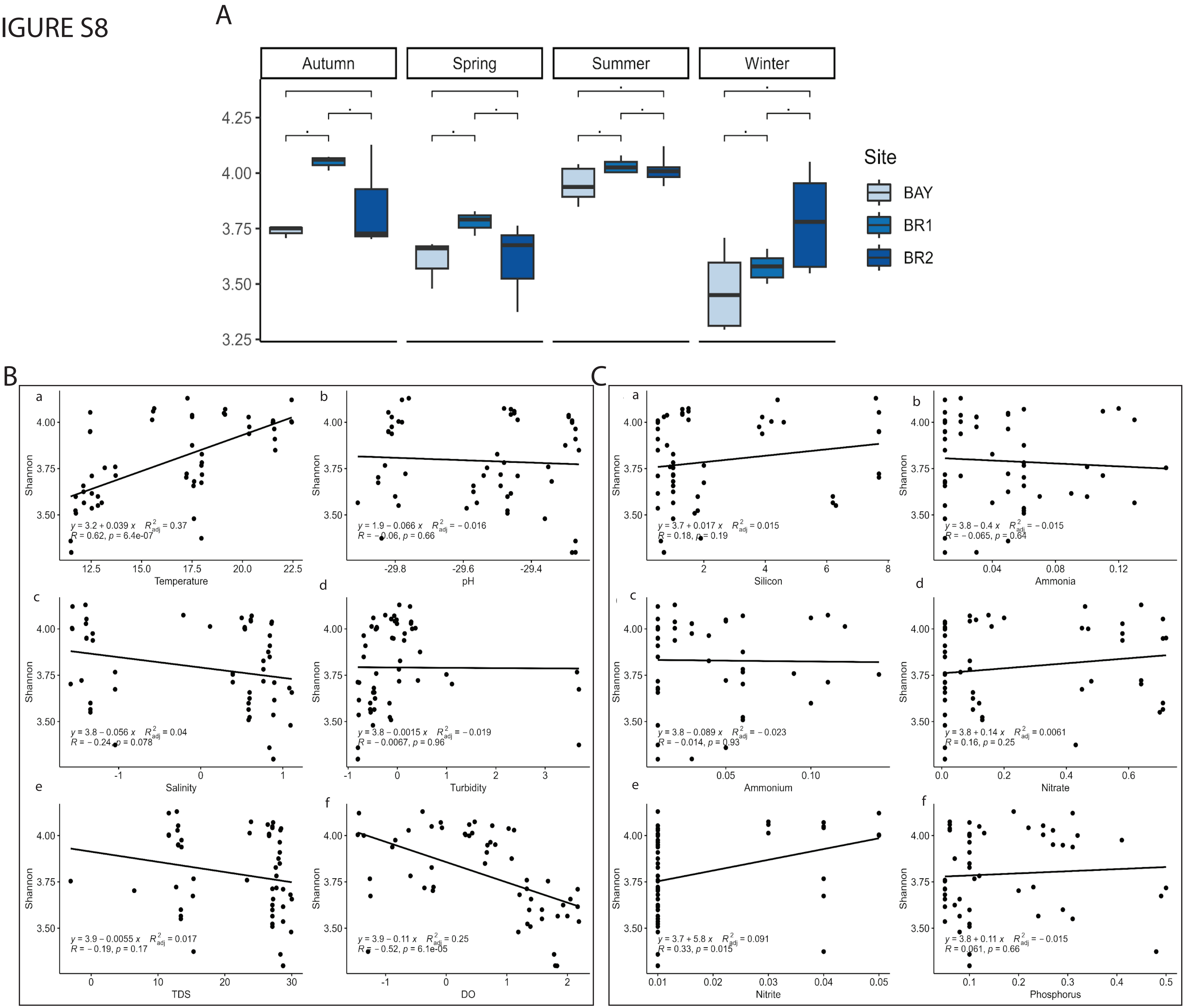

Figure S8 Correlations between alpha diversity and physicochemical variables. A)Linear regression plots between prokaryotic alpha diversity are shown in B) for physical factors (a-f) and C) for chemical factors (a-f).

FIGURE S9

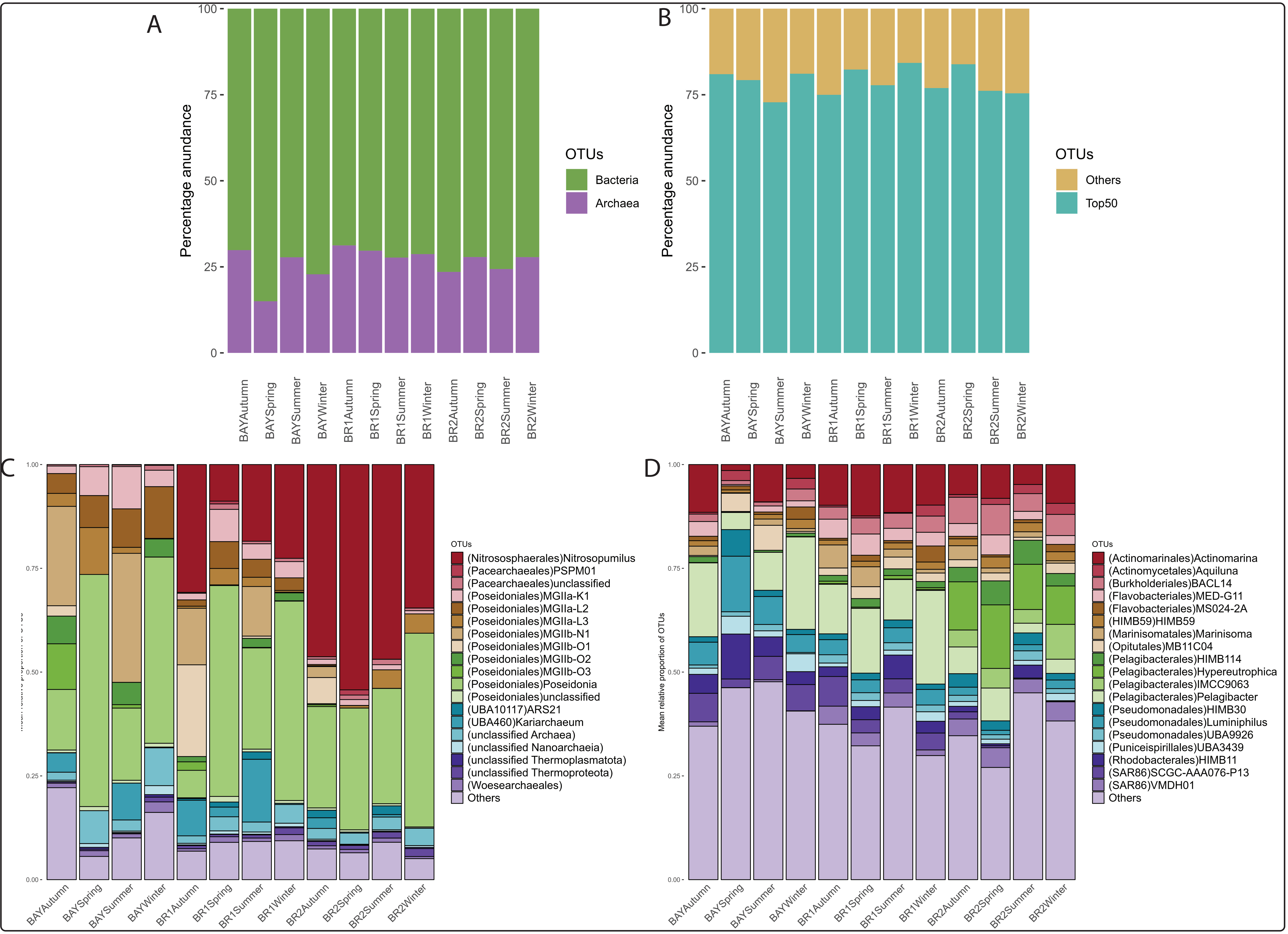

Figure S9. Relative abundance of archaea and bacteria, most abundant OTUS (Top50) and hierarchical clustering between samples A) Barplot represents relative abundances of Archaea, and Bacteria across sites and seasons. B) Plot of relative abundances between most abundant 50 OTUs (top 50 OTUs) and the rest grouped into “Others” across all samples. Barplot represents most abundant C) Archaea and D) Bacteria, respectively, and the rest are grouped into “Others” which account for 5-20% of the community.

FIGURE S10

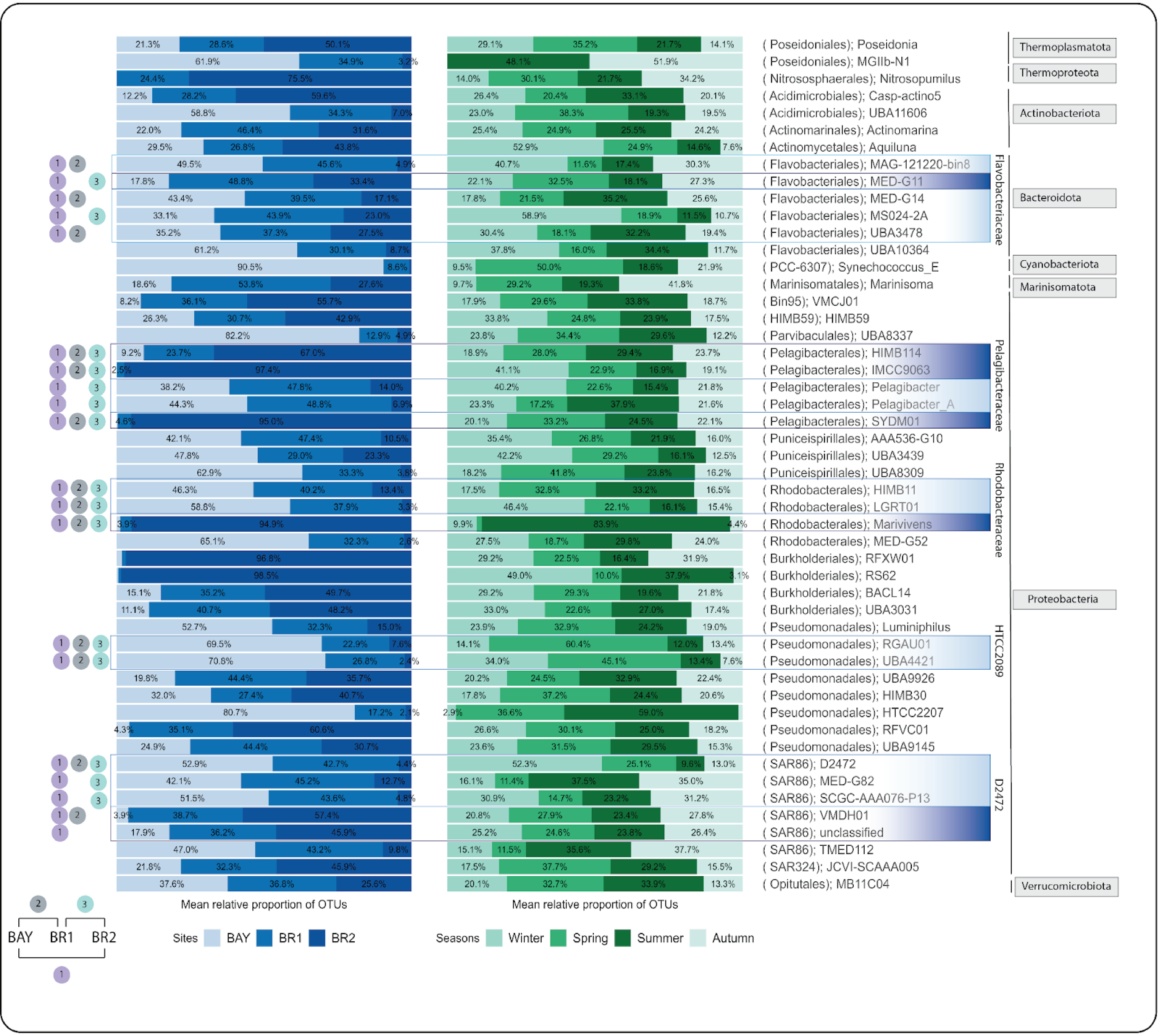

Figure S10 Initial community profile displaying trends of the 50 most abundant genera in the Brisbane River estuary using singleM. Shown are the 50 most abundant OTUs across sites and seasons, defined at the genus level, cumulatively representing >77% of the community by relative abundance. Mean relative proportions of OTUs across sites (blue shades) and seasons (green shades) were calculated for each of the 50 OTUs. For each genus level OTU, the higher ranks phylum and order are provided in bold on the right and in brackets to the left of the genus name, respectively. Genera with significantly higher abundances in the Bay (BAY) and the Brisbane River (BR1, BR2) are highlighted in coloured circled numbers, on the left. Selected genera, which were classified as marine or brackish ecotypes based on abundance comparisons between the sites BAY and BR2, are highlighted in shades of light blue (marine ecotypes) and dark blue (brackish ecotypes).

FIGURE S11

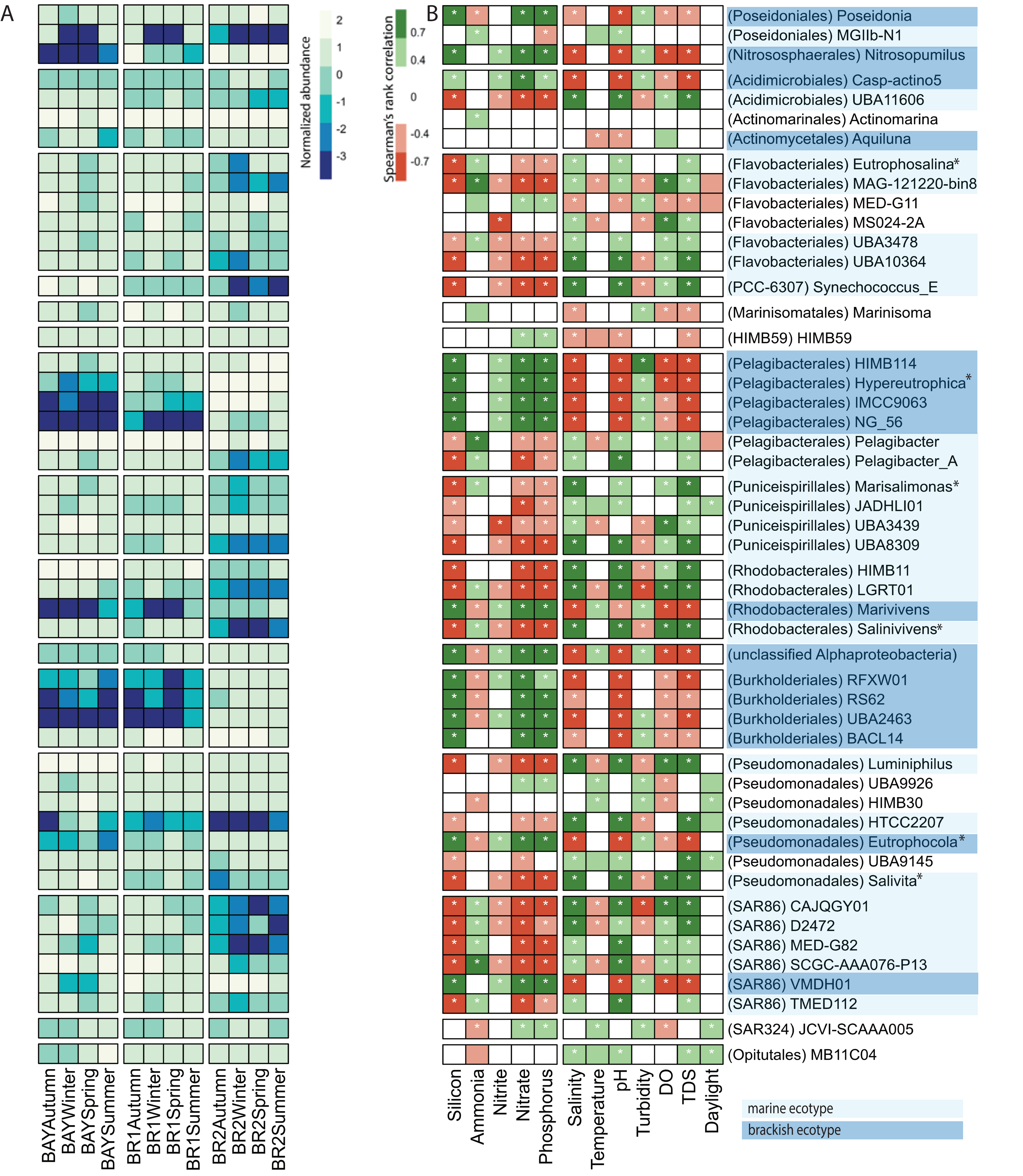

Figure S11. Community profile of 50 abundant OTUs and correlations with physico-chemical parameters. Metagenome profiling using singleM revealed 50 abundant OTUs (A) which represent >75% community by abundance, and their correlation with few physico-chemical parameters (B).

FIGURE S12

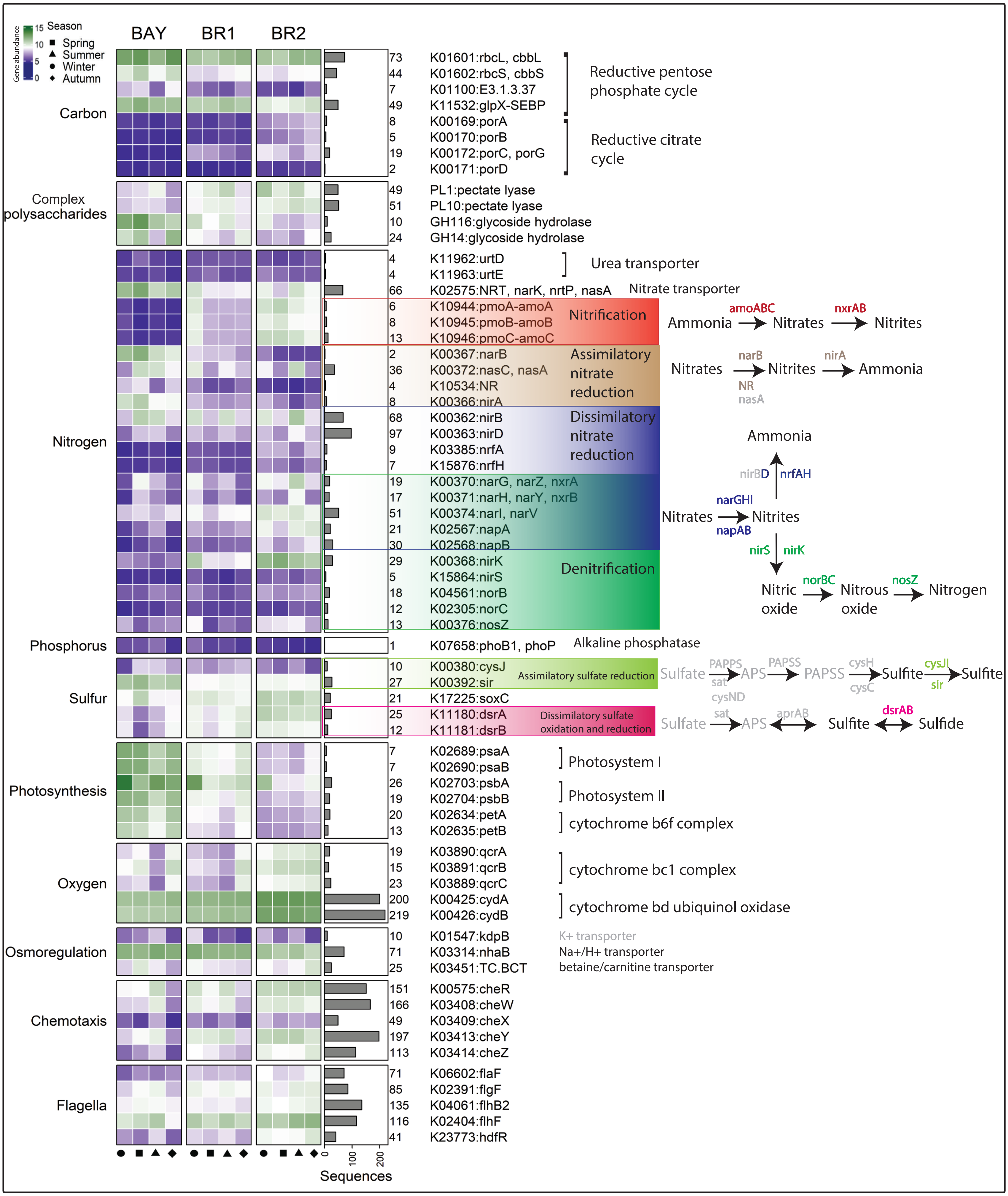

Figure S12 Gene centric analyses of genes with significantly different abundances between the brackish and marine microbial communities. Heatmap displays community abundance of differentially abundant genes identified between the sites BAY and BR2, annotated using kegg/CAZY, grouped by pathways. Bar plot on the right of the heatmap indicates the number of sequences in the gene catalog, and is an approximation of the diversity within the samples. On the far right, nitrogen and sulfur pathways are shown with genes encoding enzymes that mediate these pathways. Grey indicates pathways that contain genes that are not differentially abundant between BAY and BR2, but present in the gene catalog.

FIGURE S13

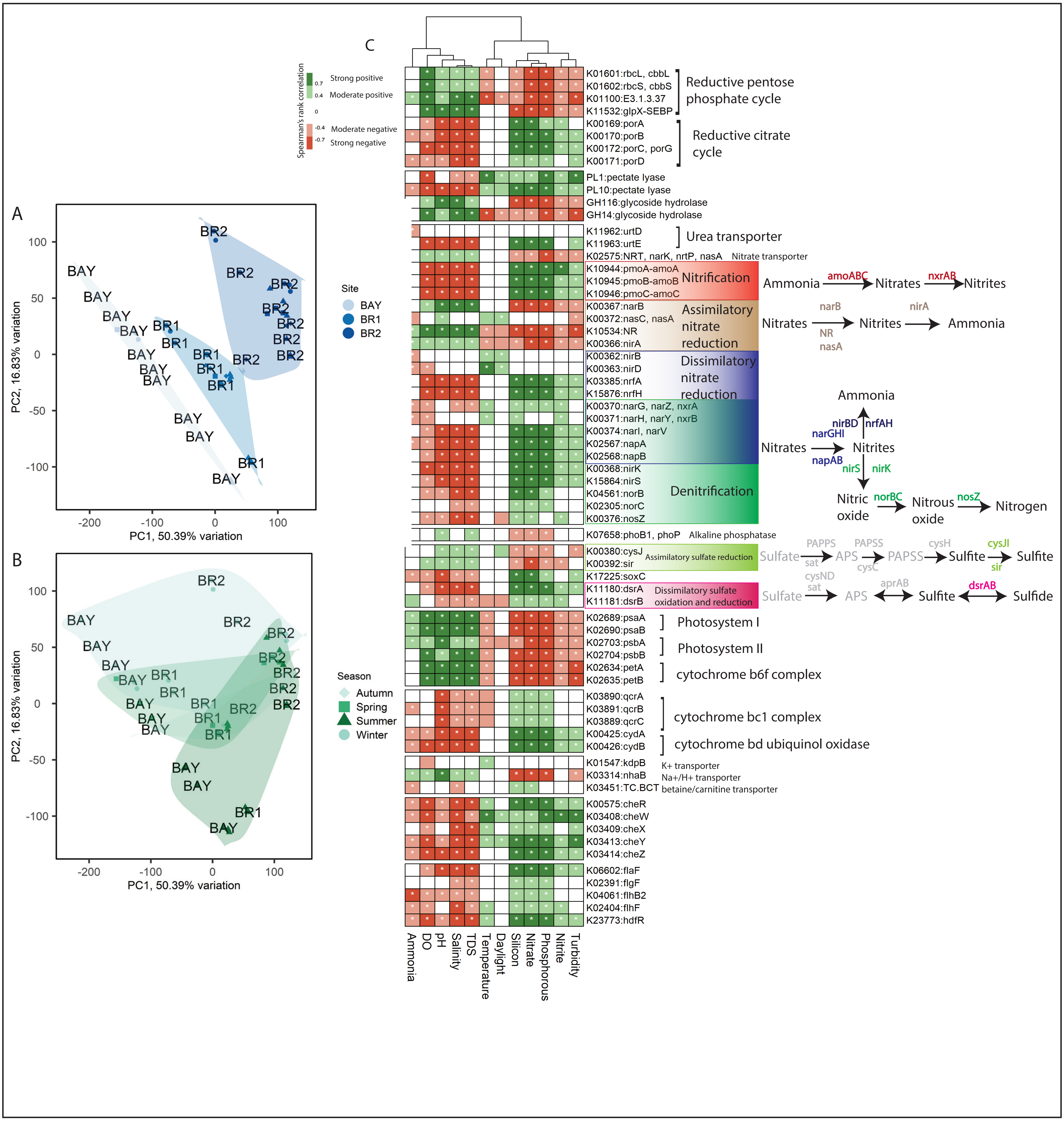

Figure S13. PCA variation plot depicting functional architecture and correlations between physico-chemical metadata against significant genes in metabolic profile of brackish-bay communities. A) and B) PCA showing community dissimilarity across sites (blue shades and seasons (green shades) C) Heatmap depicts correlations given in green shade (positive) and red shades (negative) between gene abundance and physico-chemical parameters. Genes shown are gene abundances, identified to be differentially abundant across the three sites in the study. Details on KEGG IDs are provided in Suppl. Table S9. Strength of correlation is given next to the figure legend.

FIGURE S14

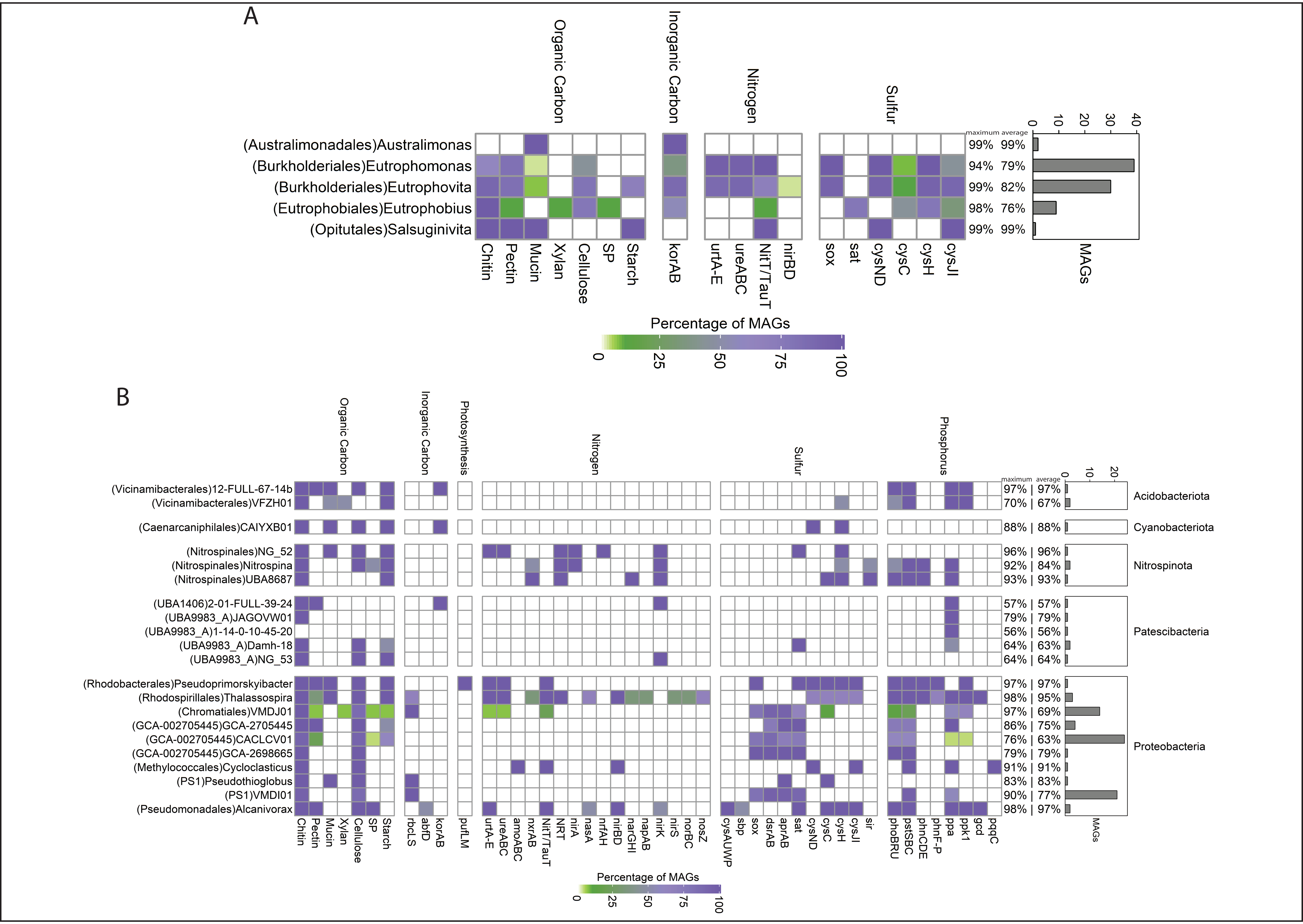

Figure S14 Metabolic profile of low abundance, newly named and rare taxa. Heatmap displays the metabolic profile of A) newly named taxa, other than those in the top 50, showing the percentage of MAGs that encode genes involved in biogeochemical cycling. The percentage on the right indicates maximum and average completeness for all MAGs associated with a genus separated by a bar "|". The barplot represents the number of MAGs in each genus. . B) displays the metabolic profile rare taxa (<0.1% abundance in community profile) in showing the percentage of MAGs that encode genes involved in biogeochemical cycling The percentage on the right indicates maximum and average completeness for all MAGs associated with a genus separated by a bar "|". The barplot represents the number of MAGs in each genus.
