# Supplementary material for: Machine learning and metagenomics identifies uncharacterized taxa inferred to drive biogeochemical cycles in a subtropical hypereutrophic estuary
Source: ISME Commun. 2024 May 10;4(1):ycae067. doi: 10.1093/ismeco/ycae067 (PMC11758582; doi:10.1093/ismeco/ycae067)
Supplement: Supplementary_Text_June2023_docx_ycae067 [file supplementary_text_june2023_docx_ycae067.pdf]

Introduction:

The **Brisbane River** (*Maiwar*) is the longest river in South-East Queensland, Australia, traversing approximately 344 km from the catchment to the estuary, where it discharges into Moreton Bay (*Quandamooka*)[8]. Average annual discharge volumes are high (1.4 x 10^6 m^3) compared to the volume of the estuary (133 x 10^6 m^3), indicating **a strong riverine impact on the estuary** [9]. However, meso-tidal forces also shape this ecosystem, carrying saltwater 80 km upstream from the river mouth which results in a long estuarine turbidity maxima zone (ETM) zone of 20–60 km **(Fig. 1A)** [9,10]. In addition, the estuary undergoes strong temporal changes influenced by higher flow rates during the wet season in summer (7.7m^3/s), compared to lower rates (3.2m^3/s) during the dry season in winter [10]. Long water residence times, ranging from 100 to 120 days, are characteristic for the Brisbane River estuary [11], and are known to be reliable indicators of effective microbial nutrient and carbon processing [12]. Traversing agricultural and urban areas, the Brisbane River has come under considerable anthropogenic pressure. **Nutrient levels** have increased considerably, resulting in high export rates of phosphorus (685 kt/year) and nitrogen (3,162 t/year), which are estimated to be 5.3 and 2.7 times greater than before European settlement [13]. Decades of dredging have increased the impact of tidal currents that resuspend **fine-grained sediment (**~4 um median particle size) from the river bed, causing a markedly higher turbidity [14,15] that is responsible for the murky, brown color of the estuary and the high sediment export rates, averaging 247 kt/year, into Moreton Bay [14]. Given the current state of the Brisbane River estuary, this waterway represents a model system to study microbial community dynamics in an anthropogenically altered, subtropical, hyper-eutrophic, estuary.

Materials and methods

Modified phenol chloroform DNA extraction method

The filters were defrosted from storage at -20°C after sampling, and parafilmed at both ends during incubation periods. To one end of the filter, 1.8 ml of lysis buffer (5 ml of 1M Tris HCl pH 8.0, 8ml 0.5M EDTA pH 8.0, 25.6 g of Sucrose, and the volume made up to 100 ml with MilliQ Water) and 18ul of Lysozyme (100mg/ml) were added, and the filter incubated in a 50 ml falcon tube in a slow shaking water bath at 37°C for 1 hour. 200ul of Proteinase K (2mg/ml) was then added and the filter incubated in a 50ml falcon tube in a slow shaking water bath at 55°C for 1 hour. The lysate was recovered in multiple 2ml tubes, where equal volumes of Phenol:Chloroform:Isoamyl alcohol were added. The tubes were then centrifuged at 16,000g for 10 minutes, and the aqueous layer (supernatant) was transferred to another tube, where equal volumes of Chloroform:Isoamyl alcohol were added. The tubes were then centrifuged at 16,000g for 10 minutes, and the aqueous layer (supernatant) was transferred to another tube, and the DNA was precipitated from the supernatant by incubating with 600ul of Isopropanol at room temperature (RT) for 15 minutes. The tubes were then centrifuged for a further 30 minutes at 4°C, which yielded a tiny white pellet, which was washed with 500ul of 70% Ethanol and centrifuged at RT for 10 minutes at 20,000g. The tubes were air dried,

and 20ul of sterilized MilliQ water was added to the pellet, and incubated at 4°C for an hour, while the DNA started to dissolve. Freeze the samples in -20°C until sample submission for metagenomic sequencing.

Metagenomic sequencing and analyses

For Oxford Nanopore library preparation and sequencing, A single library was prepared following a genomic DNA by ligation protocol (Oxford Nanopore, LSK-109). On completion of the library prep, the library was quantified using the Quant-iT™ dsDNA HS Assay Kit (Invitrogen), and QC was performed using the Agilent Genomic DNA screentapes (#5067-5365) on the TapeStation 4200 (Agilent # G2991AA) as per the manufacturer's protocol. 41 fmol of library was sequenced on a PromethION 24 (Oxford Nanopore) for a total of 72 hours on a R9.4 flow cell using MinKNOW (v20.06.18) using the default settings.

Preparing hybrid assemblies with long and short reads

Further, Nanopore sequencing yielded 40-70 Gbps of data that was basecalled using Guppy (v4.0.11), and assembled using Flye v2.4.2.3 (https://github.com/fenderglass/Flye) and then the resulting contigs were polished with Racon 1.3.2 (https://github.com/isovic/racon). The contigs were further polished with Illumina short reads using Pilon v1.22 (https://github.com/broadinstitute/pilon; [15]) followed by another polish with Racon. The corresponding short reads were then mapped to the long read polished contigs with a coverage >20X and length > 200kb with Minimap2 v2.15 (https://github.com/lh3/minimap2), and unmapped reads were along with long and short reads to create a hybrid assembly with slamM assembly and binning pipeline (https://github.com/Ecogenomics/slamM). The assembly coverage and binning was performed using MetaBAT2 v2.12.1 using the jgi_summarize_bam_contig_depths tool. The long and short reads associated with the bins were re-assembled using Unicycler v0.4.7-0 (https://github.com/rrwick/Unicycler), which were combined with high-quality and polished Flye contigs. These combined assemblies were binned with Metabat2 v2.12.1 to generate MAGs. MAG quality was determined using CheckM v1.0.13 and taxonomy was assigned using GTDB-Tk v0.3.0.

Community profile analyses

OTU abundance was calculated using the singleM 'pipe' (-p) flag, which produced a single OTU table containing trimmed mean coverage across each lineage, calculated across all genes. The data was normalized by multiplying each coverage value with respective markers (i.e. 37 for bacterial and 35 for archaeal), and normalized with DESEQ2 to carry out differential analyses between samples.

Definition of brackish and marine ecotypes

The representatives per genus were selected by filtering out genomes with a quality score (CheckM completeness - 5*CheckM contamination) of <50, <50% of the 122 archaeal marker genes, an N50 <4 Kb, >2500 contigs, or >1500 scaffolds. From the remaining genomes, the highest quality genome was selected giving preference to i) complete NCBI reference genome, ii) NCBI reference genomes, iii) complete NCBI

representative genomes, iv) NCBI representative genomes, and v) GTDB representative genomes. The supplementary table of family rank representatives are provided (see Supplementary text - family rank representatives).

Feature selection for machine learning:

Boruta is a wrapper selection algorithm around random forest. It relies on feature importance reported by random forest inherently. Since Boruta is more superior and works with random forest, it makes it better for feature selection than SelectKBest as a statistical feature selection method which relies on ANOVA F-value. For KNN, Boruta does not work, and a feature selection is required to level the playing field between 2 algorithms. Hence, the choice of feature selection and algorithm pair.

We apply a combination of metabolic reconstruction and machine learning to identify ecological microbial indicators for hypereutrophication in the estuary, e.g. nitrates and total phosphorus concentrations. The advantage over other traditional statistical methods, is that it is versatile to use and includes an ensemble of decision trees. Using IndVal to identify significant ecological indicators and Boruta to narrow down features for machine learning models, we are able to discover and explain our results which builds trust for the ML classifier chosen (ie categories for nitrates and total phosphorus). We did not proceed with regression, as it would need more data points to work with. Taking this comment into consideration, we have added this sentence in the introduction "Here, we apply a genome-focused metagenomics approach, supported by supervised machine learning, to dissect microbial communities in the subtropical Brisbane River estuary. This study is a first attempt to identify significant ecological indicator taxa using Indicator value analyses (IndVal) and random forest ML classifier. We combine this approach with the recovery of genomes from these taxa to explore their key metabolic features and infer direct and indirect responses to changes in nutrient levels in the estuary" In addition to this, while there are both advantages and disadvantages with LEfSe, traditionally it has been computed for 2 classes at a time, hence we replaced this method with a more streamlined 3-class IndVal and ML models. There can be many approaches or methods to the same problem.

Regression Analysis for Nitrate and Total phosphorus concentrations:

This section covers the regression model trained on relative OTU abundances to predict the nitrate and total phosphorus concentrations. Preprocessing and prefiltering of the data is exactly the same as described in the methods for the classification model. Instead of classification machine learning models, we used linear regression and random forest regressor machine learning methods. Table 1 shows the outcomes that we have achieved.

Here is our high level regression workflow:

1. OTU pre-filter strategy and Indicator value analyses (IndVal) to identify significant indicators is the same as performed for classification strategy.
2. We performed repeated fold cross validation to get the r2 scores.
3. Model training: Three models were used, a Linear regression and Random forest (boruta for feature selection).
4. Calculated the cross validation R2 score.

Table 1: R2 cross validation score for nitrate and total phosphorus.

Concentrations	Linear regression R2 score	Random forest R2 score
Nitrate	-0.753	-0.087
Total Phosphorus	-30.503	0.309

All scores reported in Table 1 are cross validation R2 scores. Cross validation is performed using repeated fold cross validation with fold of 5 and repeat of 5.

We aimed to stratify and test the R2 score again. Stratification helps to preserve the underlying distribution of the target variable. The scores improved compared to Table 1 (except for Linear regression in case of total phosphorus) but still reported poor results. All scores reported in Table 2 are cross validation R2 scores after stratification.

Table 2: R2 cross validation score (after stratification) for nitrate and total phosphorus.

Concentrations	Linear regression R2 score	Random forest R2 score
Nitrate	-0.0145	0.77
Total Phosphorus	-79.52	0.56

Below, we provide the code snippets without stratification:

Nitrate

```
[ ] # repeated kfold cross validation using linear regression
    cv_kfold = RepeatedKFold(n_splits = 5, n_repeats = 5, random_state = 1)
    cv_model_pipeline = Pipeline([('vt' , VarianceThreshold()), ('scale', StandardScaler()),('lr', LinearRegression())])

    # evaluate model
    scores = cross_val_score(cv_model_pipeline, otu_data_file_t_abundant.loc[:, otu_data_file_t_abundant.columns != 'target'] , otu_data_file_t_abundant['target'], scoring='r2', cv=cv_kfold, n_jobs=-1)
    print(scores)

    # report performance
    print('R2 Score: %.3f (%.3f)' % (mean(scores), std(scores)))

    [-1.15006396e-01  4.36986495e-01  2.55335070e-01  5.46984295e-01
      5.94875273e-01 -9.72440332e-03  5.92680427e-01  2.70833124e-02
      9.93500204e-02  7.89545326e-01  2.99689805e-01  8.68724697e-02
     -2.72370200e+01  4.71606293e-01  6.46108070e-01  4.95216959e-01
      4.42426454e-01  7.54231398e-01  2.89451427e-01  4.23593510e-01
      5.67638601e-01  3.46644194e-01  6.68464421e-01  2.12893858e-01
     -5.13534609e-01]
    R2 Score: -0.753 (5.414)
```

```
    # repeated kfold for random forest regression
    cv_kfold = RepeatedKFold(n_splits = 5, n_repeats = 5, random_state = 1)
    cv_model_pipeline = Pipeline([('vt' , VarianceThreshold()), ('bt',  BorutaPy(verbose=0,estimator=RandomForestRegressor(random_state=4))), ('rd', RandomForestRegressor(random_state=4))])

    # evaluate model
    scores = cross_val_score(cv_model_pipeline, otu_data_file_t_abundant.loc[:, cols_otu] , otu_data_file_t_abundant['target'], scoring='r2', cv=cv_kfold, n_jobs=-1)
    print(scores)

    # report performance
    print('R2 Score: %.3f (%.3f)' % (mean(scores), std(scores)))

    [ 1.36376766e-02  8.84740425e-01  9.87824853e-01  7.28137815e-01
      9.19557109e-01  5.99853839e-01  9.60441819e-01  6.66179021e-01
      6.06231603e-01  8.87686800e-01  8.68845400e-01  4.71347520e-01
     -2.00812942e+01  9.30951393e-01  8.85128465e-01  7.47816694e-01
      6.23873734e-01  8.56373577e-01  8.04841217e-01  7.78294295e-01
      6.95758114e-01  6.80436602e-01  8.68261069e-01  9.17741257e-01
      5.17993806e-01]
    R2 Score: -0.087 (4.086)
```

Total phosphorus

```
[10] # repeated kfold cross validation using linear regression
     cv_kfold = RepeatedKFold(n_splits = 5, n_repeats = 5, random_state = 1)
     cv_model_pipeline = Pipeline([('vt' , VarianceThreshold()), ('scale', StandardScaler()),('lr', LinearRegression())])

     # evaluate model
     scores = cross_val_score(cv_model_pipeline, otu_data_file_t_abundant.loc[:, otu_data_file_t_abundant.columns != 'target'] , otu_data_file_t_abundant['target'], scoring='r2', cv=cv_kfold, n_jobs=-1)
     print(scores)

     # report performance
     print('R2 Score: %.3f (%.3f)' % (mean(scores), std(scores)))

     [  -1.29917161  -13.90111577  -26.64551885 -106.4398181   -31.69365784
        -8.51378424  -28.33878436   -8.5475193   -12.55011804  -35.63368915
       -11.14146999  -14.67641235  -25.44597927 -153.99086997   -6.20514517
        -2.90333965  -51.32876321  -22.22209399  -33.04154705   -1.23998222
       -72.89437568  -33.69059271  -45.7623029    -9.77415437   -4.69903878]
     R2 Score: -30.503 (34.631)
```

3.2 train - random forest

```
[12] # repeated kfold for random forest regression
     cv_kfold = RepeatedKFold(n_splits = 5, n_repeats = 5, random_state = 1)
     cv_model_pipeline = Pipeline([('vt' , VarianceThreshold()), ('bt',  BorutaPy(verbose=0,estimator=RandomForestRegressor(random_state=4))), ('rd', RandomForestRegressor(random_state=4))])

     # evaluate model
     scores = cross_val_score(cv_model_pipeline, otu_data_file_t_abundant.loc[:, otu_data_file_t_abundant.columns != 'target'] , otu_data_file_t_abundant['target'], scoring='r2', cv=cv_kfold, n_jobs=-1)
     print(scores)

     # report performance
     print('R2 Score: %.3f (%.3f)' % (mean(scores), std(scores)))

     [-0.04905896  0.62914868  0.67493983  0.65476541  0.82994055  0.37435668
       0.63824247  0.71525094 -1.21079624  0.7082419   0.86700165  0.2656942
      -2.57643998  0.50328623 -0.66479458  0.32890342  0.54568078  0.58241645
       0.63110332  0.57148227  0.83576528 -0.00863264  0.79858648  0.7199175
       0.34938188]
     R2 Score: 0.309 (0.754)
```

Below, we provide the code snippets with stratification:

Nitrates: Linear regression

```
[ ]  # kfold for cross validation r2 score
     folds = 5
     avg_fold_r2_score = []

     # cross validation using linear regression
     cv_model_pipeline = Pipeline([('vt' , VarianceThreshold()), ('scale', StandardScaler()),('lr', LinearRegression())])
     cv_kfold = create_folds(otu_data_file_t_abundant, n_s=folds, n_grp=1000)

     for f in range(0,folds):
       X_train = cv_kfold[cv_kfold['Fold'] != f].loc[:,train_cols ]
       y_train = cv_kfold[cv_kfold['Fold'] != f]['target']

       X_test = cv_kfold[cv_kfold['Fold'] == f].loc[:,train_cols ]
       y_test = cv_kfold[cv_kfold['Fold'] == f]['target']

       fold_r2_score = cv_model_pipeline.fit(X_train,y_train).score(X_test, y_test)
       print(f"Current fold {f} r2 score:{fold_r2_score}")
       avg_fold_r2_score.append(fold_r2_score)

     print(f"Average fold r2 score:{sum(avg_fold_r2_score) / folds}")
     print(f"Std deviation of r2 score:{statistics.stdev(avg_fold_r2_score)}")

     Current fold 0 r2 score:0.329018840109236
     Current fold 1 r2 score:-1.137633033158385
     Current fold 2 r2 score:0.3235430915038928
     Current fold 3 r2 score:0.14964162502100364
     Current fold 4 r2 score:0.26291615307140515
     Average fold r2 score:-0.014502664690569489
     Std deviation of r2 score:0.6319826600487208
     /usr/local/lib/python3.10/dist-packages/sklearn/model_selection/_split.py:700: UserWarning: The least populated class in y has only 1 members, which is less than n_splits=5.
       warnings.warn(
```

Nitrates: Random forest regression

```
[ ] # kfold for cross validation r2 score
    folds = 5
    avg_fold_r2_score = []
    # cross validation using linear regression
    cv_model_pipeline = Pipeline([('vt' , VarianceThreshold()), ('bt',  BorutaPy(verbose=0,estimator=RandomForestRegressor(random_state=4))), ('rd', RandomForestRegressor(random_state=4))])
    cv_kfold = create_folds(otu_data_file_t_abundant, n_s=folds, n_grp=1000)

    for f in range(0,folds):
      X_train = cv_kfold[cv_kfold['Fold'] != f].loc[:,train_cols ]
      y_train = cv_kfold[cv_kfold['Fold'] != f]['target']

      X_test = cv_kfold[cv_kfold['Fold'] == f].loc[:,train_cols ]
      y_test = cv_kfold[cv_kfold['Fold'] == f]['target']

      fold_r2_score = cv_model_pipeline.fit(X_train.loc[:,train_cols ],y_train).score(X_test.loc[:, train_cols], y_test)
      print(f"Current fold {f} r2 score:{fold_r2_score}")
      avg_fold_r2_score.append(fold_r2_score)


    print(f"Average fold r2 score:{sum(avg_fold_r2_score) / folds}")
    print(f"Std deviation of r2 score:{statistics.stdev(avg_fold_r2_score)}")
```

```
/usr/local/lib/python3.10/dist-packages/sklearn/model_selection/_split.py:700: UserWarning: The least populated class in y has only 1 members, which is less than n_splits=5.
  warnings.warn(
Current fold 0 r2 score:0.7309979275436138
Current fold 1 r2 score:0.6813921390100022
Current fold 2 r2 score:0.9359914535113097
Current fold 3 r2 score:0.7072523404941171
Current fold 4 r2 score:0.837865401053827
Average fold r2 score:0.778699852322574
Std deviation of r2 score:0.10617229111849676
```

Total Phosphorus:Linear regression

```
[ ] # kfold for cross validation r2 score
    folds = 5
    avg_fold_r2_score = []
    # cross validation using linear regression
    cv_model_pipeline = Pipeline([('vt' , VarianceThreshold()), ('scale', StandardScaler()),('lr', LinearRegression())])
    cv_kfold = create_folds(otu_data_file_t_abundant, n_s=folds, n_grp=1000)

    for f in range(0,folds):
      X_train = cv_kfold[cv_kfold['Fold'] != f].loc[:,train_cols ]
      y_train = cv_kfold[cv_kfold['Fold'] != f]['target']

      X_test = cv_kfold[cv_kfold['Fold'] == f].loc[:,train_cols ]
      y_test = cv_kfold[cv_kfold['Fold'] == f]['target']

      fold_r2_score = cv_model_pipeline.fit(X_train,y_train).score(X_test, y_test)
      print(f"Current fold {f} r2 score:{fold_r2_score}")
      avg_fold_r2_score.append(fold_r2_score)

    print(f"Average fold r2 score:{sum(avg_fold_r2_score) / folds}")
    print(f"Std deviation of r2 score:{statistics.stdev(avg_fold_r2_score)}")
```

```
Current fold 0 r2 score:-3.071574152251001
Current fold 1 r2 score:-359.8368011128652
Current fold 2 r2 score:-12.720645363008556
Current fold 3 r2 score:-15.744225026396027
Current fold 4 r2 score:-6.249629606020172
Average fold r2 score:-79.52457505210819
Std deviation of r2 score:156.78003789319465
/usr/local/lib/python3.10/dist-packages/sklearn/model_selection/_split.py:700: UserWarning: The least populated class in y has only 1 members, which is less than n_splits=5.
  warnings.warn(
```

Total Phosphorus: Random forest regression

```
[ ] # kfold for cross validation r2 score
    folds = 5
    avg_fold_r2_score = []
    # cross validation using linear regression
    cv_model_pipeline = Pipeline([('vt' , VarianceThreshold()), ('bt',  BorutaPy(verbose=0,estimator=RandomForestRegressor(random_state=4))), ('rd', RandomForestRegressor(random_state=4))])
    cv_kfold = create_folds(otu_data_file_t_abundant, n_s=folds, n_grp=1000)

    for f in range(0,folds):
      X_train = cv_kfold[cv_kfold['Fold'] != f].loc[:,train_cols ]
      y_train = cv_kfold[cv_kfold['Fold'] != f]['target']

      X_test = cv_kfold[cv_kfold['Fold'] == f].loc[:,train_cols ]
      y_test = cv_kfold[cv_kfold['Fold'] == f]['target']

      fold_r2_score = cv_model_pipeline.fit(X_train,y_train).score(X_test, y_test)
      print(f"Current fold {f} r2 score:{fold_r2_score}")
      avg_fold_r2_score.append(fold_r2_score)


    print(f"Average fold r2 score:{sum(avg_fold_r2_score) / folds}")
    print(f"Std deviation of r2 score:{statistics.stdev(avg_fold_r2_score)}")
```

```
/usr/local/lib/python3.10/dist-packages/sklearn/model_selection/_split.py:700: UserWarning: The least populated class in y has only 1 members, which is less than n_splits=5.
  warnings.warn(
Current fold 0 r2 score:0.7597818738458543
Current fold 1 r2 score:0.5384644515380159
Current fold 2 r2 score:0.31612400887685144
Current fold 3 r2 score:0.6860916613007076
Current fold 4 r2 score:0.5155068048445508
Average fold r2 score:0.563193760081196
Std deviation of r2 score:0.17152246715711136
```

Results:

Preliminary analyses into predictor variables nitrate and total phosphorus for ML workflow:

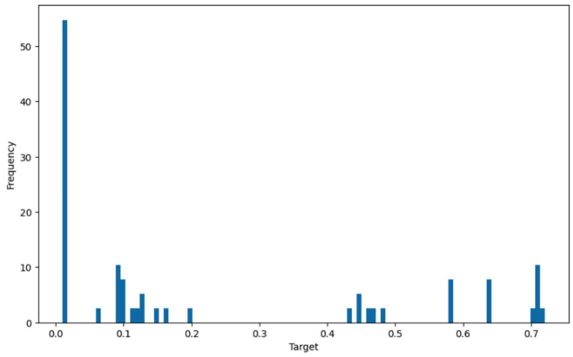

Figure 1: Histogram of nitrate concentration values

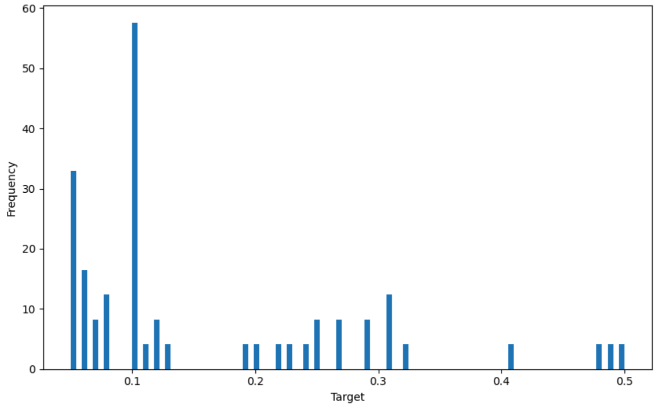

Figure 2: Histogram of total phosphorus concentration values

Differential gene abundance patterns reveal community niche differentiations along environmental gradients

Applying a gene centric approach, we evaluated the functional capabilities of the prokaryotic community across sites and seasons. For this task, we compiled a gene catalogue containing approximately 5.2 million non-redundant genes, of which 2.8 million genes (54%) could be annotated (**Table supplementary text**). Gene profiles mainly separated by site and to a lower extent by season, in particular at site BR2 (**Fig. S7A, B**). The most pronounced differences in relative gene abundances were observed between the sites BAY and BR2 (**Fig. 4, Table S9,10**). The BAY was characterised by a significantly higher abundance of genes encoding pathways for carbon fixation, i.e. the reductive pentose phosphate cycle (*rbc, SEBP*), complex polysaccharide degradation (GH116, GH14), assimilatory nitrate reduction (ANR), and sulphate reduction (ASR) **(Fig. 4, Table S9,10)**. Genes encoding enzymes involved in photosynthesis, i.e. photosystem I and II (*psa, psb*), the cytochrome b6f complex (*pet*), and Na+/H+ transporters for osmoregulation (*nhaB*) were also significantly enriched at the BAY. Brackish communities at site BR2 were characterised by significantly higher relative abundances of inferred enzymes involved in nitrification (*amoABC*), betaine/carnitine

transporters for osmoregulation (*TC.BCT*), chemotaxis (*cheRWXYZ*), and the flagellar assembly (*flaF, flgF, flhF, flhB2, hdfR*) **(Fig. 4, Table S9,10)**.

The association between gene abundances and environmental parameters revealed that metabolic genes were significantly correlated with nutrients such as nitrate, nitrite, phosphorus and physical parameters including DO, pH, salinity, and temperature (**Fig. S7**). For example, nitrification genes (*amoABC*) and several nitrogen removal genes (*napAB, nirKS, norBC, nosZ*) had a strong positive correlation with nitrate and nitrite concentrations. ANR genes (NR, *nirA*) had a strong negative correlation with nitrate, and a moderate positive correlation with ammonia. Genes involved in the reductive pentose phosphate cycle, oxidative phosphorylation, chemotaxis and flagellar assembly showed a strong positive correlation with phosphorus. Reductive pentose phosphate cycle (rbcLS), photosystem I and II (psa, psb) and complex polysaccharide degradation genes (GH116, GH14) had a strong positive correlation with DO, pH, and salinity. Several genes associated with complex polysaccharide degradation (pectins: PL1, PL10), chemotaxis (cheW) and in DNRA (nirBD) were positively correlated with temperature.

Osmoregulation and flagellar system of metagenome-assembled genomes

Most abundant taxa were inferred to employ strategies to overcome osmotic stress in the hyper-eutrophic estuary, through uptake of organic solutes, such as glycine betaine/proline transporter (*proXV*), and/or adaptations to higher salt concentrations such as potassium transporters *(trkA)* and Na+ translocating dehydrogenases (*nqrF*) (**Fig. 6, Fig. S13**). In addition, most Pseudomonadales genera encode the Na+/H+ antiporter (*nhaC*), and two genera (HTCC2207, UBA4582) of this order carry a betaine/carnitine transporter gene (*TC.BCT*) (**Fig. 6, Fig. S13**). Motility might be a common feature among estuary bacteria since many taxa, including Rhodobacterales and Pseudomonadales genera, encode a flagellar system (*fliGMN, motAB*). A complete set of chemotaxis genes (cheBR, cheAW, cheY) was identified in the genera JCVI-SCAAA005 (order SAR324), UBA9659 and *Luminiphilus* (Pseudomonadales), as well as in *Marivivens* (order Rhodobacterales).

Proposed new taxa

We propose 12 species and 11 genera based on the MAGs recovered from this study. Based on those 11 novel genera, we further propose eight families and three orders, and their description is provided below.

Description of *Eutrophosalina marina gen. nov., sp. nov.*

Eu.tro.pho.sa.li'na Gr. adv. eû good; Gr. adj. trophos, feeder; N.L. masc. adj. salinus, salty; N.L. fem. n. Eutrophosalina, a microbe adapted to eutrophic and saline environments and ma.ri'na L. fem. adj. marina, of the sea. Type species of the genus is *Eutrophosalina marina*. The former name for this genus is MED-G14. Inferred to be marine ecotype (Table S16), and can break down a wide range of organic carbon substrates (Chitin, Pectin, Mucin, Cellulose, Sulfated polysaccharides and Starch). Also carries a polyphosphate kinase gene for polyphosphate accumulation, likely fuelling cytochrome c oxidase (coxABCD). Can perform

osmoregulation using trk1 (K+ transporter) and nqrF (Na+ transporter). Based on the genome reporting standards for MAGs, the estimated completeness was 96.32%, contamination 0%, and the presence of the 16S (1,522 bp), and 23S (2,775 bp) rRNA gene and 34 tRNAs . The type genome is defined as "high-quality" draft MAG (NCBI Accession : JAYQTD000000000) , having two contigs and a genome size of 1.5Mbps.

Description of *Australimonas brisbanensis gen. nov., sp. nov.*

Aus.tra.li.mo'na.s L. adj. australis, southern; N.L. fem. n. monas, a unit; N.L. fem. n. Australimonas, a unit from the south, referring to Australia where the organism was found and bris.ban.en'sis N.L. fem. adj. brisbanensis, of or belonging to Brisbane. Type species of the genus is *Australimonas brisbanensis*. This is a novel group of the order and family UBA2963. Inferred to degrade Mucin, and carry genes for phosphate and phosphonate transporters. Can perform osmoregulation using trkA (K+ transporter) and multicomponent Na+/H+ transportation (mnh). Based on the genome reporting standards for MAGs, the estimated completeness was 98.68%, contamination 0%, and the presence of the 16S (1,472 bp), and 23S (2,898 bp) rRNA gene and 46 tRNAs .The type genome is defined as "high-quality" draft MAG (NCBI Accession : JAYQTA000000000) , and a genome size of 1.9Mbps

Description of *Hypereutrophica brisbanensis gen. nov., sp. nov.*

Hi.pe.reu.tro.phi.ca Gr. prep. hyper above; Gr. adv. eû good; Gr. masc. adj. trophicos nursing, feeding; N.L. fem. n. N.L. adj. Hypereutrophica, a bacterium found in hyper-eutrophic environments and bris.ban.en'sis N.L. fem. adj. brisbanensis, of or belonging to Brisbane. Type species of the genus is *Hypereutrophica brisbanensis*. The former name for this genus is SYDM01. Inferred to break down a wide range of organic carbon substrates (Chitin, Pectin, and Cellulose). Few MAGs carry abfD gene inferred to take part in 3-Hydroxypropionate bi-cyle. Can likely break down urea (ureABC) and can take up nitrates (NRT2). Based on the genome reporting standards for MAGs , the estimated completeness was 91.57%, contamination 7.09%, and the presence of the 16S (1,468 bp), and 23S (2,679 bp) rRNA gene and 26 tRNAs . The type genome is defined as "high-quality" draft MAG (NCBI Accession :JAYQTL000000000) , and a genome size of 0.9Mbps

Description of *Marisalimonas marina gen. nov., sp. nov.*

Ma.ri.sa.li.mo'na.s L. neut. n. mare, the sea; L. mas. n. sal, salt; N.L. fem. n. monas, a unit; N.L. masc. n. Marisalimonas, a microbe occurring in saline conditions in the sea and ma.ri'na L. fem. adj. marina, of the sea. Type species of the genus is *Marisalimonas marina*. The former name for this genus is AAA536-G10. Inferred to break down a wide range of organic carbon substrates (Chitin, Pectin, Mucin and Starch) as well as fix inorganic carbon via the Calvin cyle (rbcLS). Capable of nitrate (NRT2), sulphate (ssu) and phosphorus uptake (pst, pho) and regulation, as well as osmoregulate via multiple genes (trkA, nqrF, mnh, proXV). Could have possible motility using motAB and fliGMN genes. Based on the genome reporting standards for MAGs , the estimated completeness 91.69%, contamination 0.14%, and the presence of the 5S

(109 bp), 16S (1,482 bp), and 23S (2,712 bp) rRNA gene and 32 tRNAs . The type genome is defined as "high-quality" draft MAG (NCBI Accession : JAYPPH000000000) ,with six contigs, genome size of 1.9Mbps.

Description of *Salinivivens marinus gen. nov., sp. nov.*

Sa.li.ni.vi'vens N.L. masc. adj. salinus, salty; L. part. adj. vivens, living; N.L. masc. n. Salinivivens a microbial organism thriving in saline conditions and ma.ri'nus L. masc. adj. marinus, of the sea. Type species of the genus is Salinivivens marinus. The former name for this genus is MED-G52. Inferred to break down a wide range of organic carbon substrates (Chitin, Pectin, Cellulose and Starch). Capable of performing aerobic anoxygenic photosynthesis (pufLM), and thiosulfate oxidation (soxABCDXYZ), likely performing osmoregulation using trk1 (K+ transporter) and proXV (Glycine betaine) and inferred to be motile (fliGMN, motAB gene). Also carries genes for phosphorus uptake and regulation(pho, pst). Based on the genome reporting standards for MAGs , the estimated completeness 94.41%, contamination 0.3%, and the presence of the 5S (108 bp), 16S (1,457 bp), and 23S (2, 718bp) rRNA gene and 32 tRNAs .The type genome is defined as "high-quality" draft MAG (NCBI Accession : JAYQKI000000000) , with genome size of 2.6 Mbps.

Description of *Eutrophomonas brisbanensis gen. nov., sp. nov.*

Eu.tro.pho.mo'na.s Gr. adv. eû good; Gr. adj. trophos, feeder; L. fem. n . monas, a unit N.L. fem. n. Eutrophomonas, a unit associated with eutrophic environments and bris.ban.en'sis N.L. fem. adj. brisbanensis, of or belonging to Brisbane. Type species of the genus is *Eutrophomonas brisbanensis*.This is a novel group of the family UBA3031 within order Burkholderiales. Inferred to break down a wide range of organic carbon substrates (Chitin, Pectin, Cellulose and Starch) as well as carries genes for rTCA cycle to fix inorganic carbon (korABCD). Encodes genes for urea uptake and degradation (ureABC, urtABCDE), phosphorus uptake and regulation (pho, pst), pyrroquinoline quinone (pqqC), nitrate uptake (NRT2), thiosulfate oxidation (sox) and sulphate assimilation (cys). There is noted absence of osmoregulation genes. Based on the genome reporting standards for MAGs , the estimated completeness 98.78%, contamination 0%, and the presence of the 16S (1,532 bp), and 23S (2, 933 bp) rRNA gene and 40 tRNAs . The type genome is defined as "high-quality" draft MAG (NCBI Accession : JAYQIQ000000000) , with one contig and genome size of 2.1 Mbps.

Description of *Eutrophovita brisbanensis gen. nov., sp. nov.*

Eu.tro.ph.o.mo'nas. Gr. adv. eû good; Gr. adj. trophos, feeder; L. fem. n. monas, a unitN.L. masc. n. Eutrophovita, a unit associated with eutrophic environments, and bris.ban.en'sis N.L. fem. adj. brisbanensis, of or belonging to Brisbane. Type species of the genus is *Eutrophovita brisbanensis*. This is a novel group of the family UBA3031 within order Burkholderiales. Inferred to break down a wide range of organic carbon substrates (Chitin, Pectin, Cellulose and Starch) as well as carries genes for rTCA cycle to fix

inorganic carbon (korABCD). Encodes genes for urea uptake and degradation (ureABC, urtABCDE), phosphorus uptake and regulation (pho, pst), polyphosphate kinase (ppk1), pyrroquinoline quinone (pqqC), thiosulfate oxidation (sox) and sulphate assimilation (cys). Based on the genome reporting standards for MAGs , the estimated completeness 92.77%, contamination 0.61%, and the presence of the 16S (1,532 bp), and 23S (2,933 bp) rRNA gene and 37 tRNAs . The type genome is defined as "high-quality" draft MAG (NCBI Accession : JAYQTM000000000) ,with genome size of 2.1 Mbps.

Description of *Salivita marina gen. nov., sp. nov.*

Sa.li.vi.ta L. mas. n. sal, salt; L. fem. n. vita, life; N.L. fem. n. Salivita, an organism associated with saltwater, and ma.ri'na L. fem. adj. marina, of the sea. Type species of the genus is *Salivita marina*. The former name for this genus is RGAU01. Inferred to break down a wide range of organic carbon substrates (Chitin, Mucin, Xylan, Cellulose and Starch) as well as carries genes for rTCA cycle to fix inorganic carbon (korABCD). Encodes genes for nitrate uptake (NRT2), thiosulfate oxidation (soxABCDXYZ), sulphate assimilation (cys), phosphorus uptake and regulation (pho, pst), polyphosphate kinase and phosphatase (ppa). Can perform osmoregulation (trkA, nqrF, nhaC) and is possibly motile (fliGMN, motAB). Based on the genome reporting standards for MAGs , the estimated completeness was 91.02%, contamination 1.36%, and the presence of the 5S (110bp), 16S (1,532 bp), and 23S (2,880 bp) rRNA gene and 42 tRNAs . The type genome is defined as "high-quality" draft MAG (NCBI Accession :JAYQNS000000000) , with genome size of 3.3Mbps.

Description of *Eutrophocola salsuginis gen. nov.*

Eu.tro.pho.a.co'la Gr. adv. eû good; Gr. adj. trophos, feeder; L. masc./fem. suff. -cola inhabitant of; N.L. fem. n. Eutrophocola, referring to a microbe in eutrophic aquatic environments in Queensland, and sal.su'gi.nis L. gen. n. salsuginis, of the brackish water. Type species of the genus is *Eutrophocola salsuginis*. The former name for this genus is RFVC01. Inferred to break down a Chitin and Pectin, as well as carries genes for rTCA cycle to fix inorganic carbon (korABCD). Carries genes for nitrate uptake (NRT2) and anaerobic dissimilatory nitrate reduction (nirBD). Capable of sulfate assimilation (cys), phosphorus uptake and regulation (pho, pst) and polyphosphate accumulation (ppk1). Likely performing osmoregulation using trk1 (K+ transporter) and proXV (Glycine betaine) and inferred to be motile (fliGMN, motAB gene). Based on the genome reporting standards for MAGs , the estimated completeness was 93.58%, contamination 1.79%, and the presence of the 5S (106bp), 16S (1,551 bp), and 23S (2,877 bp) rRNA gene and 42 tRNAs . The type genome is defined as "high-quality" draft MAG (NCBI Accession : JAYQTB000000000) ,with genome size of 3Mbps.

Description of *Eutrophobius brisbanensis gen. nov., sp. nov.*

Eu.tro.pho.bi'us Gr. adv. eû good; Gr. adj. trophos, feeder; Gr. masc. n. bios, life; N.L. masc. n. Eutrophobius, life associated with eutrophic environments, and bris.ban.en'sis N.L. masc. adj. brisbanensis,

of or belonging to Brisbane. The type species of the genus is *Eutrophobius brisbanensis*. This is a novel group of the family and order UBA4486. Inferred to break down a Chitin and Cellulose as well as carries genes for rTCA cycle to fix inorganic carbon (korABCD). Likely performing sulfate assimilation (cys), polyphosphate accumulation (ppk1) and osmoregulation (trkA, nqrF, mnh). Based on the genome reporting standards for MAGs , the estimated completeness was 98.24%, contamination 0.88%, and the presence of the 5S (110bp), 16S (1.540 bp), and 23S (2,877 bp) rRNA gene and 46 tRNAs . The type genome is defined as “high-quality” draft MAG (NCBI Accession : JAYPQE000000000) ,with genome size of 2.7Mbps.

Description of *Nitrosopumilus brisbanensis sp. nov.*

brisbanensis, of or belonging to Brisbane. The former name for this species is *Nitrosopumilus* sp008080815. Based on the genome reporting standards for MAGs , the estimated completeness was 98.63%, contamination 0%, and the presence of the %S (111 bp), 16S (1,464 bp), and 23S (3,117 bp) rRNA gene and 43 tRNAs . The type genome is defined as “high-quality” draft MAG (NCBI Accession : JAYOXR000000000) ,with genome size of 1.3Mbps.

Description of *Salsuginivita brisbanensis gen. nov., sp. nov.*

Sal.su.gi.ni.vi’ta L. fem. n. salsugo, brackish water; N.L. fem. n. vita, life; N.L. fem. n. Salsuginivita, a bacterium found in brackish habitats, and bris.ban.en'sis N.L. fem. adj. brisbanensis, of or belonging to Brisbane. Type species of the genus is *Salsuginivita brisbanensis*. The former name for this genus is CAIYFD01. Inferred to break down a wide range of organic carbon substrates (Chitin, Pectin, Mucin, Starch) as well as carries genes for rTCA cycle to fix inorganic carbon (korABCD). Capable of nitrate uptake (NRT2), phosphorus uptake and regulation (pho, pst), polyphosphate accumulation (ppk1) and noted absence of osmoregulation genes. Based on the genome reporting standards for MAGs , the estimated completeness was 98.63%, contamination 0%, and the presence of the 16S (1,555 bp), and 23S (2, 882bp) rRNA gene and 46 tRNAs. The type genome is defined as “high-quality” draft MAG (NCBI Accession :JAYQOU000000000) ,with genome size of 2.5Mbps.

Description of *Australimonadaceae* fam. nov.

Aus.tra.li.mo.na.da.ce'ae (N.L. fem. n. Australimonas type genus of the family; -aceae ending to denote a family; N.L. fem. pl. n. Australimonadaceae the Australimonas family). The description for *Australimonadaceae* is the same for *Australimonas*. The former name for this family is UBA2963.

Description of Australimonadales ord. nov.

Aus.tra.li.mo.na.da'les (N.L. fem. n. Australimonas type genus of the order; -ales ending to denote an order; N.L. fem. pl. n. Australimonadales the Australimonas order. The description for Australimonadales is same for *Australimonas.* The former name for this order is UBA2963.

Description of Marisalimonadaceae fam. nov

Mari.sa.li.mo.na.da.ce'ae (N.L. fem. n. Marisalimonas type genus of the family; -aceae ending to denote a family; N.L. fem. pl. n. Marisalimonadaceae the Marisalimonas family). The description for Marisalimonadaceae is the same for *Marisalimonas*. The former name for this family is AAA536-G10.

Description of Eutrophomonadaceae fam. nov.

Eu.tro.pho.mo.na.da.ce'ae (N.L. fem. n. Eutrophomonas type genus of the family; -aceae ending to denote a family; N.L. fem. pl. n. Eutrophomonadaceae the Eutrophomonas family). The description for Eutrophomonadaceae is the same for *Eutrophomonas*. The former name for this family is UBA3031.

Description of Eutrophovitaceae fam. nov.

Eu.tro.pho.vi.ta.ce'ae (N.L. fem. n. Eutrophovita type genus of the family; -aceae ending to denote a family; N.L. fem. pl. n. Eutrophovitaceae the Eutrophovita family). The description for Eutrophovitaceae is same for *Eutrophovita*. The former name for this family is UBA3031.

Description of Salivitaceae fam. nov.

Sa.li.vi.ta.ce'ae (N.L. fem. n. Salivita type genus of the family; -aceae ending to denote a family; N.L. fem. pl. n. Salivitaceae the Salivita family). The description for Salivitaceae is the same for *Salivita*. The former name for this family is HTCC2089.

Description of Eutrophobiaceae fam. nov.

Eu.tro.pho.bi.a.ce'ae (N.L. masc. n. Eutrophobius type genus of the family; -aceae ending to denote a family; N.L. fem. pl. n. Eutrophobiaceae the Eutrophobius family). The description for Eutrophobiaceae is same for *Eutrophobius*. The former name for this family is UBA4486.

Description of Eutrophobiales fam. nov.

Eu.tro.pho.bi.a'les. (N.L. masc. n. Eutrophobius type genus of the order; -ales ending to denote an order; N.L. fem. pl. n. Eutrophobiales the Eutrophobius order). The description for Eutrophobiales is same for *Eutrophobius*. The former name for this order is UBA4486.
